# RNA Modification of N6-Methyladenosine Predicts Immune Phenotypes and Therapeutic Opportunities in Kidney Renal Clear Cell Carcinoma

**DOI:** 10.3389/fonc.2021.642159

**Published:** 2021-03-18

**Authors:** Huihuang Li, Jiao Hu, Anze Yu, Belaydi Othmane, Tao Guo, Jinhui Liu, Chunliang Cheng, Jinbo Chen, Xiongbing Zu

**Affiliations:** ^1^Department of Urology, Xiangya Hospital, Central South University, Changsha, China; ^2^Immunobiology & Transplant Science Center, Houston Methodist Research Institute, Texas Medical Center, Houston, TX, United States

**Keywords:** kidney renal clear cell carcinoma, N6-methyladenosine, immune phenotype, immune checkpoint blockade, tumor microenvironment

## Abstract

RNA modification of N6-methyladenosine (m6A) plays critical roles in various biological processes, such as cancer development, inflammation, and the anticancer immune response. However, the role played by a comprehensive m6A modification pattern in regulating anticancer immunity in kidney renal clear cell carcinoma (KIRC) has not been fully elucidated. In this study, we identified two independent m6A modification patterns with distinct biological functions, immunological characteristics, and prognoses in KIRC. Next, we developed an m6A score algorithm to quantify an individual's m6A modification pattern, which was independently validated in external cohorts. The m6A cluster 1 and low m6A score groups were characterized by a hot tumor microenvironment with an increased infiltration level of cytotoxic immune cells, higher tumor mutation burden, higher immune checkpoint expression, and decreased stroma-associated signature enrichment. In general, the m6A cluster 1 and low m6A score groups reflected an inflammatory phenotype, which may be more sensitive to anticancer immunotherapy. The m6A cluster 2 and high m6A score groups indicated a non-inflammatory phenotype, which may not be sensitive to immunotherapy but rather to targeted therapy. In this study, we first identified m6A clusters and m6A scores to elucidate immune phenotypes and to predict the prognosis and immunotherapy response in KIRC, which can guide urologists for making more precise clinical decisions.

## Introduction

Kidney renal clear cell carcinoma (KIRC) is a common urinary cancer with increasing incidence (1). Despite advances in targeted therapy, the prognosis of patients with advanced KIRC remains extremely poor (1). The emergence of anticancer immune checkpoint blockade (ICB) therapy has revolutionized the treatment of advanced KIRC and significantly improved survival status (2–4). However, response rates to ICB in advanced KIRC are low, even though KIRC is an immunogenic cancer characterized by a high tumor mutation burden (TMB) (5). These low response rates indicated that there were some primary or secondary resistance mechanisms to ICB. Hence, to decrease adverse events and economic burden and identify the best candidates to receive ICB treatment, it is necessary to explore these resistance mechanisms and identify reliable predictors for response to ICB response.

RNA modification of N6-methyladenosine (m6A) is the most prominent and abundant RNA modification pattern in eukaryotic cells (6). M6A modification is a dynamically reversible process regulated by methyltransferases (writers), demethylases (erasers), and binding proteins (readers) (6, 7). Moreover, it plays a critical role in various biological processes, such as cancer occurrence, progression and inflammation (8, 9). Recently, m6A modification has been found to play an essential role in anticancer immune regulation (10). Wang et al. elucidated that depletion of METTL3/14 promoted secretion of IFN-γ, CXCL9, and CXCL10, subsequently inducing infiltration of CD8+ T cells, which overcomes resistance to ICB (11). In contrast, another study reported that METTL3 activates dendritic cells by increasing m6A levels of CD40, CD80, and TLR4, priming cytotoxic T lymphocyte activation (12). Interestingly, the same m6A writer gene (METTL3) exerted the opposite role in regulating anticancer immunity. FTO, an m6A eraser gene, promoted tumor immune evasion by increasing expression of immune checkpoint genes, such as LILRB4 and PD-1 (13, 14). Genetic depletion or pharmacological inhibition of FTO reactivates immune surveillance and overcomes resistance to ICB. Furthermore, Han et al. revealed the potential of YTHDF1 as a promising therapeutic target in anticancer immunotherapy (15). They demonstrated that genetic depletion of YTHDF1 significantly enhanced tumor antigen cross-presentation and CD8+ T cell priming. Therefore, m6A modification represents a potential emerging immunotherapy target and predictor of response to ICB response.

However, all of the studies above are confined to only one or two m6A modification genes because of technical limitations. As we all known, antitumor effect and tumor microenvironment (TME) can be regulated by numerous factors (16). Therefore, comprehensive analysis of multiple m6A regulators will improve our understanding of antitumor effect and TME. In this study, we comprehensively analyzed m6A modification patterns based on 24 m6A genes in KIRC. To the best of our knowledge, the number of m6A genes included in this manuscript is the largest reported to date. Additionally, we correlated m6A modification patterns with the immune phenotype and response to ICB for the first time.

## Materials and Methods

**Figure 1:**
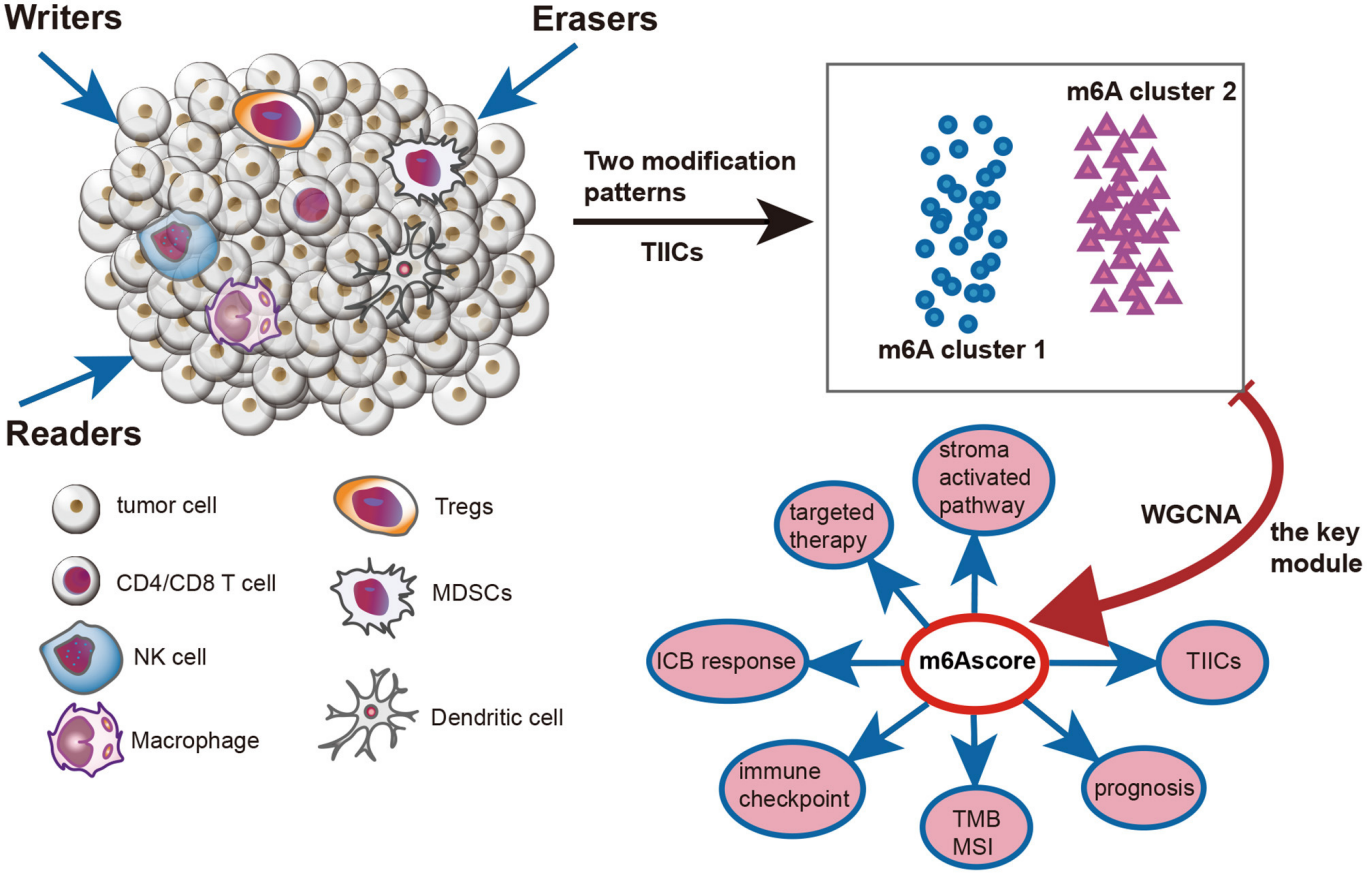
Mechanism diagram of this study.

Figure 1 illustrates the mechanism diagram of our study and Supplementary Figure 1 shows the workflow of our study.

### Data Retrieval and Preprocessing

#### Cancer Genome Altas (TCGA) Data

RNA sequencing data (FPKM value), mutation profiles, and clinical data for TCGA-KIRC were downloaded from the Genomic Data Commons (GDC, https://portal.gdc.cancer.gov/) using the R package TCGAbiolinks (17). The FPKM value was transformed into transcripts per kilobase million (TPM) value. After removing duplicated patients, we included 530 KIRC patients with full clinical information and 72 normal tissues for further analysis. The copy number variation (CNV) data, processed with the GISTIC algorithm, were downloaded from the UCSC Xena data portal (http://xena.ucsc.edu/). Somatic mutation data were analyzed using VarScan2 and used to calculate the tumor mutation burden (TMB). Microsatellite instability (MSI) data were collected from the supplementary files of Bonneville's study (18).

#### Other Data Sources

A KIRC cohort (GSE22541) with detailed survival data and an RNA expression matrix was downloaded from GEO (https://www.ncbi.nlm.nih.gov/geo/). After removing 44 samples collected from pulmonary metastasis of KIRC, we included 24 samples collected from primary KIRC for further analysis. An immunotherapy cohort (PMID29301960) containing 33 KIRC patients was collected from the supplementary files of Miao's study (19). Based on the Creative Commons 3.0 License, an immunotherapy cohort (IMvigor210) containing 348 bladder cancer patients was obtained from http://research-pub.gene.com/IMvigor210CoreBiologies/ (20). Another immunotherapy cohort of melanoma (GSE78220) was downloaded from GEO. After removing one duplicated patient and one patient without follow-up time, we included 26 patients of GSE78220 for further analysis.

Detailed information on these cohorts is summarized in Supplementary Table 1.

### Unsupervised Clustering for 24 m6A Regulator Genes

We systematically identified 24 m6A regulator genes in our study from previous studies (16, 21). These m6A genes included eight writers (METTL3, METTL14, RBM15, RBM15B, WTAP, KIAA1429, CBLL1, and ZC3H13), two erasers (ALKBH5 and FTO), and 14 readers (YTHDC1, YTHDC2, YTHDF1, YTHDF2, YTHDF3, IGF2BP1, IGF2BP2, IGF2BP3, HNRNPA2B1, HNRNPC, FMR1, LRPPRC, ELAVL1, and EIF3A). Unsupervised clustering analysis was then conducted to comprehensively identify differential m6A modification patterns using the ConsensuClusterPlus package (22). Finally, the TCGA-KIRC cohort was classified into several clusters with different biological functions using a consensus clustering algorithm.

### Functional Analysis Between Different m6A Clusters

First, we downloaded 50 hallmark pathways from the MSigDB database (23). These 50 pathways systematically reflect the majority of the biological functions of humans. The GSVA algorithm was applied to calculate the enrichment scores of these pathways using the “GSVA” R package (24). Then, we analyzed difference in these pathways between different m6A clusters using the LIMMA algorithm (25). An adjusted *P* < 0.05 was considered statistically significant. Second, the limma R package's empirical Bayesian approach was applied to determine differentially expressed genes (DEGs) between different m6A clusters. The significance criteria for determining DEGs were set as an adjusted *P* < 0.05 and |logFC|>1. Finally, we performed Gene Ontology (GO) and Kyoto Encyclopedia of Genes and Genomes (KEGG) analyses using the ClusterProfiler R package based on these DEGs.

### Depicting Immunological Characteristics of the TME in KIRC

The anticancer immune response, also called the cancer immunity cycle, is composed of seven key steps in the TME: the release and presentation of cancer cell antigens (Steps 1 and 2), the priming and activation of the immune system (Step 3), trafficking and infiltration of immune cells into tumors (Steps 4 and 5), and recognition and killing of cancer cells by T cells (Steps 6 and 7) (26). The activities of these seven steps were downloaded from http://biocc.hrbmu.edu.cn/TIP/ (27). Then, the single-sample gene-set enrichment analysis (ssGSEA) algorithm was used to quantify the relative abundance of tumor-infiltrating immune cells (TIICs) in the TME based on specific immune cell gene sets obtained from the study of Charoentong (Supplementary Table 2) (28). In addition, to avoid calculation errors caused by different algorithms and mark gene sets, we validated the infiltration level of TIICs using Cibersort-ABS, xCell and TIMER algorithm (29–31).

Mariathasan et al. revealed a set of gene signatures related to immune processes and stromal pathways, such as the CD8 T-effector signature, epithelial-mesenchymal transition (EMT) markers, and the panfibroblast TGF-b response signature (Pan-FTBRS) (20). We also collected 19 gene signatures related to the clinical response to the anti-PD-L1 agent atezolizumab (Supplementary Table 3). The ssGSEA algorithm was used to calculate the enrichment score of individuals.

### Generation of Co-expression Module Networks

The R package “WGCNA” was used to develop the gene co-expression network and to identify the m6A cluster-related module (32). First, TPM data from the TCGA-KIRC dataset were tested to determine whether they were good genes or samples. Then, the filtered genes were used to calculate the connection strength and to develop a scale-free network. The gradient method was used to test the scale independence and modules' average connectivity degree. The degree of independence was set as 0.85, and then we chose a suitable power value when the connectivity degree was relatively higher (33). Next, scale-free gene co-expression networks were generated using the selected power value. A heatmap was drawn to describe the interactions between different modules and clinical characteristics, and we chose the module that had the strongest relationship with the m6A cluster.

### Generation of m6A Score

An m6A score was developed to quantify the m6A modification pattern in an individual patient with KIRC. First, we conducted univariate Cox analysis on genes of the module that had the strongest relationship with the m6A cluster and subsequently identified those genes with prognostic value. Similar to previous studies, we then performed principal component analysis (PCA) on these prognostic genes to calculate principal component 1, which was used for m6A score calculation (16, 34).

$$\begin{array}{l} m6A\;score=\;\sum PC1_{i} \end{array}$$

where i is the selected gene.

### External Validation and Drug Sensitivity Analysis

To confirm the robustness of this m6A score, we validated the prognostic value and the association between the m6A score and immunological characteristics of the TME in an independent KIRC cohort (GSE22541).

The functions significantly differed among m6A clusters. We further compared the drug sensitivities between different m6A clusters. First, we collected 184 common anticancer drugs and their target genes from the DrugBank database (www.drugbank.ca). In addition, we validated the predictive value of the m6A score for the response to ICB in three external immunotherapy cohorts.

### Statistical Analysis

Correlations between m6A regulators, m6A score and cancer immunity cycle and m6A score and pathways related to the ICB response were explored by Spearman coefficients and distance correlation analyses. Continuous variables fitting a normal distribution between binary groups were compared using a *t*-test and presented as mean ± standard deviation (SD). Otherwise, the Mann-Whitney U test was applied. Chi-square or Fisher exact tests were used to compare differences between categorical variables. The “survcutpoint” function for the maximum rank statistic was applied to determine the optimal cutoff value of the m6A score. The survival curves for prognostic analyses of categorical variables were generated using the Kaplan-Meier method, while the log-rank test was applied to estimate the statistical significance. The hazard ratio (HR) for m6A regulators was calculated using univariate Cox regression model. The independent prognostic factor of m6A score was conducted using multivariate Cox regression model and the forestplot R package was used to visualize the results. The receiver operating characteristic (ROC) curve and area under the curve (AUC) were conducted to assess the specificity and sensitivity of m6A score using time ROC R package. The mutations of m6A regulators and mutation profiles between high and low m6A score groups were visualized using maftools R package. The level of significance was set at *P* < 0.05, and all statistical tests were two-sided. Finally, all statistical data analyses were implemented using R software, version 3.6.3 (http://www.r-project.org).

## Results

### Multi-Omics Analysis of m6A Genes in KIRC

**Figure 2:**
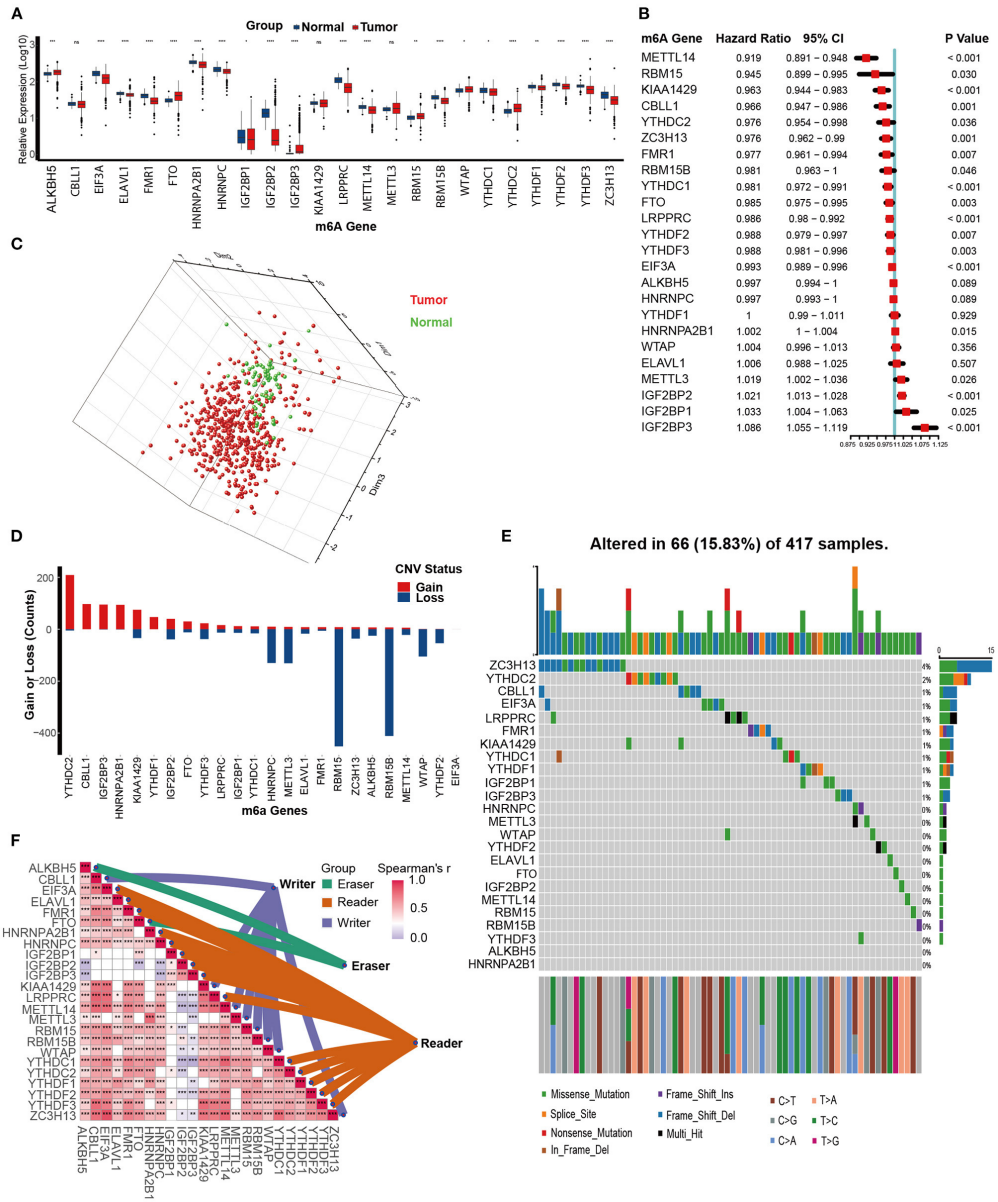
Multi-omics analysis of m6A genes in kidney renal clear cell carcinoma (KIRC). **(A)** Expression of 24 m6A genes between tumor and normal tissues in the TCGA-KIRC dataset. Tumor, red; Normal, blue. **(B)** The prognostic analyses for 24 m6A genes in the TCGA-KIRC dataset using the univariate Cox regression model. **(C)** Principal component analysis (PCA) of the expression profiles of 24 m6A genes between tumor and normal tissues in the TCGA-KIRC dataset. Tumor, red; Normal, green. **(D)** The copy number variation (CNV) frequency of 24 m6A genes in the TCGA-KIRC dataset. The height of the column represents the count, and the color represents gains or losses. Gains, red; Losses, blue. **(E)** The mutation frequency of 24 m6A genes in 417 patients with kidney clear cell carcinoma from the TCGA-KIRC cohort. Column presents individual patients. The upper bar plot represents TMB. The number on the right represents the mutation frequency in each regulator. The right bar plot represents the proportion of each variant type. The stacked bar plot below represents the fraction of conversions in each sample. **(F)** Expression correlations between 24 m6A regulators in the TCGA-KIRC dataset using Spearman analyses. Eraser, green; Reader, brown; Writer, purple (ns, Not Significant; *P < 0.05; **P < 0.01; ***P < 0.001; ****P < 0.0001).

We first analyzed the expression patterns of 24 m6A genes in KIRC and normal tissues. Interestingly, the majority of m6A writers and readers, such as METTL14, EIF3A, YTHDC1, YTHDF1, and YTHDF2, were significantly downregulated in KIRC compared to normal tissues. In contrast, expression of two m6A eraser genes (FTO and ALKBH5) was significantly higher in KIRC (Figure 2A). This expression imbalance between m6A writer and eraser genes may lead to abnormal m6A modification patterns and consequently promote the development of KIRC. Similarly, most of the m6A genes were prognostic factors. METTL14, RBM15, KIAA1429, CBLL1, YTHDC2, ZC3H13, FMR1, RBM15B, YTHDC1, FTO, LRPPRC, YTHDF2, YTHDF3, and EIF3A were favorable prognostic factors. On the other hand, METTL3, IGF2BP1, IGF2BP2, IGF2BP3, and HNRNPA2B1 were adverse prognostic factors (Figure 2B). Based on the expression of these 24 m6A genes, we could completely distinguish KIRC samples from normal samples (Figure 2C). These results suggested that m6A genes are potential diagnostic and prognostic predictors in KIRC.

Next, we assessed the CNV and mutation profiles of 24 m6A genes. Analysis of CNV data revealed prevalent CNV alterations in 24 m6A genes, and most were focused on amplification of YTHDC2, while RBM15 and RBM15B had the highest frequency of CNV deletion (Figure 2D). However, mutations of m6A genes were not frequent. Among 417 KIRC samples, only 66 (15.83%) exhibited mutations in m6A genes. ZC3H13 exhibited the highest mutation frequency at 4%, followed by YTHDC2 (2%) (Figure 2E). Finally, the close connections between the majority of m6A genes laid the foundation for the subsequent m6A clustering analysis (Figure 2F, Supplementary Table 4).

### Depicting m6A Clusters and Correlating Them With Biological Functions

**Figure 3:**
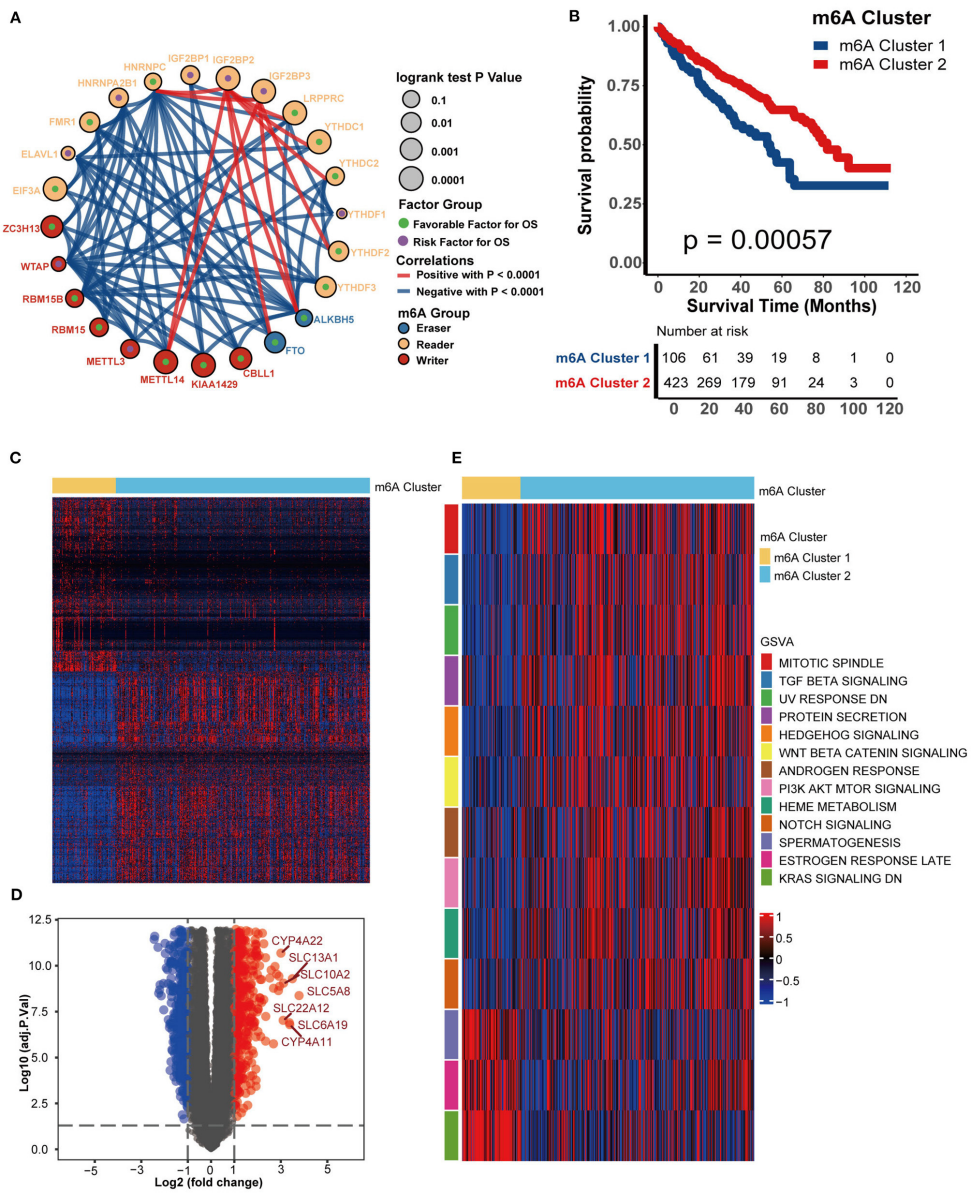
m6A modification patterns and corresponding biological characteristics. **(A)**. Correlations between 24 m6A genes in KIRC. The size of the circle represents the prognosis of each gene, and values were calculated by the log-rank test, which ranged from 0.1 to 0.0001. Green dots represent favorable factors for overall survival, while purple dots in the circle represent risk factors for overall survival. The color of the lines shows the correlation between regulators. Negative correlation, blue; Positive correlation, red. **(B)** Survival analysis for m6A clusters from the TCGA-KIRC dataset. m6A cluster 1 is shown in blue and m6A cluster 2 is shown in red. **(C)** A heatmap was drawn based on the differentially expressed genes between m6A clusters 1 and 2. Differentially expressed genes with higher expression are shown in red, and genes with lower expression are shown in blue. **(D)** A volcano plot was drawn based on the differentially expressed genes between m6A clusters 1 and 2. Differentially expressed genes with log2(fold change) higher than 1 were shown in red while the genes lower than −1 were shown in blue, and the genes without different expression were shown in gray. **(E)** GSVA analysis showed the activation (red) or inhibition (blue) of biological pathways between m6A modification patterns.

Figure 3A shows the comprehensive landscapes of 24 m6A genes concerning their prognostic value, correlations, and groups. Most of them were prognostic factors and were significantly correlated with each other, which prompted us to perform a comprehensive unsupervised clustering analysis based on these 24 m6A gene expression profiles. The results were robust when the TCGA-KIRC cohort was divided into two independent clusters. One hundred six patients were classified into m6A cluster 1, whereas the remaining 423 patients were classified into m6A cluster 2. m6A cluster 1 exhibited a significantly poorer prognosis (*P* = 0.00057) (Figure 3B). The DEGs between m6A clusters are displayed in a heatmap and volcano plot (Figures 3C,D, Supplementary Table 5). The results of GO analysis suggested that these DEGs were enriched in several biological processes, including organic anion transport, metal ion transmembrane transporter activity, collagen-containing extracellular matrix, and cellular divalent inorganic cation homeostasis (Supplementary Figures 2A–C, Supplementary Table 6). The results of KEGG analysis indicated that these DEGs were enriched in pathways such as neuroactive ligand-receptor interaction, bile secretion, vascular smooth muscle contraction, mineral absorption, complement and coagulation cascades, serotonergic synapse, protein digestion and absorption, and leukocyte transendothelial migration (Supplementary Figure 2D, Supplementary Table 7). Finally, the enrichment scores of many hallmark signatures significantly differed between the two clusters. As shown in Figure 3E, TGF-beta signaling, Wnt-beta catenin signaling, protein secretion, PI3K-Akt-Mtor signaling, androgen response, heme metabolism, mitotic spindle, and Notch signaling were enriched in m6A cluster 2. In contrast, spermatogenesis, estrogen response late, and KRAS signaling DN were enriched in m6A cluster 1 (Figure 3E, Supplementary Table 8).

### m6A Clusters Correlate With Immune Phenotypes and Immunotherapy-Related Signatures

**Figure 4:**
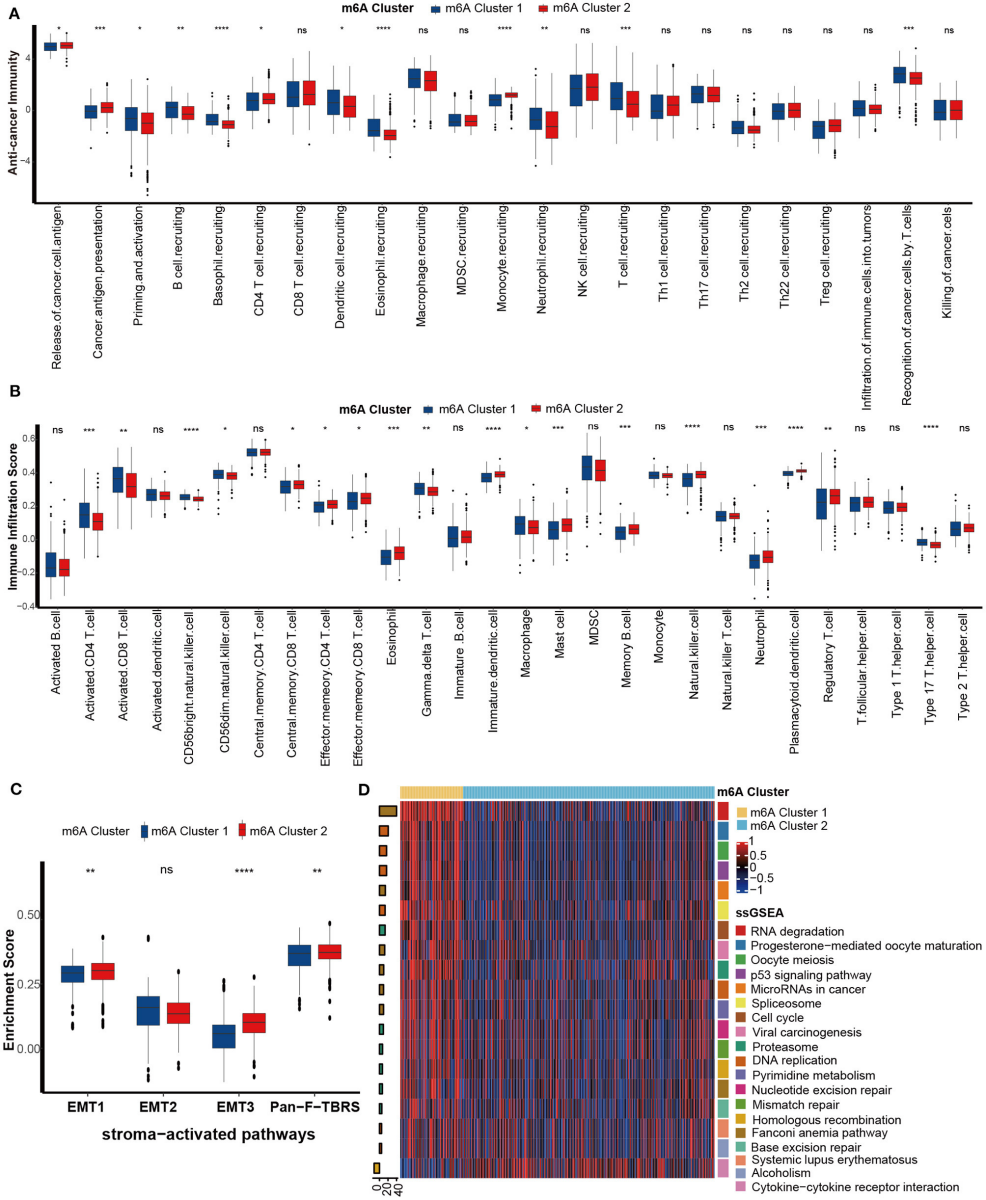
Differences in immunological characteristics between m6A clusters. **(A)** Activities of cancer immunity cycles between the two distinct m6A modification patterns. m6A cluster 1, blue; m6A cluster 2, red. **(B)** TME immune cell infiltration scores between the two distinct m6A modification patterns. m6A cluster 1, blue; m6A cluster 2, red. **(C)** Differences in stroma-activated pathways between the two distinct m6A modification patterns. m6A cluster 1, blue; m6A cluster 2, red. **(D)** Differences in immunotherapy-predicted pathways between the two m6A clusters. Left bar plots represent log10 *p-*values, red bars represent activated pathways, and blue bars represent inhibited pathways. The colors of the right bar plots represent different pathways, as shown in the legend (ns, Not Significant; *P < 0.05; **P < 0.01; ***P < 0.001; ****P < 0.0001).

We next comprehensively correlated the m6A clusters with immune phenotypes. First, we focused on the activities of anticancer immunity cycles. The activity of priming and activation of the immune system of m6A cluster 1 was significantly higher than that of m6A cluster 2, while the activities of releasing and presenting cancer cell antigens were lower (Figure 4A). In addition, the activities of T cell recruiting, B cell recruiting, and dendritic cell recruiting were consistently higher in m6A cluster 1 (Figure 4A). Finally, activities of recognition of cancer cells by T cells were higher in m6A cluster 1. To confirm these findings, we directly compared the infiltration level of tumor-infiltrating immune cells between m6A clusters. As expected, the abundance of several antitumor immune cells, such as activated CD8 T cells, activated CD4 T cells, CD56bright natural killer cells and type 17 T helper cell, was significantly higher in m6A cluster 1 than in m6A cluster 2 (Figure 4B). However, the abundance of the most recognized protumor immune cells, including regulatory T cells, immature dendritic cells, and plasmacytoid dendritic cells, was significantly downregulated in m6A cluster 1 (Figure 4B). Based on these results, we proposed that m6A cluster 1 may be an inflammatory immune phenotype, while m6A cluster 2 may be a non-inflammatory phenotype. Previous research demonstrated that stroma-associated pathways, such as EMT and Pan-FTBRS signatures, inhibited the anticancer immunity in TME (20). Here, EMT1, EMT3, and Pan-F-TBRS enrichment scores were significantly downregulated in m6A cluster 1 (Figure 4C).

Inflammatory tumor phenotypes are more sensitive to ICB (35, 36). Consistently, pathways that were positively related to the ICB response, such as RNA degradation, the cell cycle, and DNA replication, were enriched in m6A cluster 1 (inflammatory phenotype). In contrast, the pathway cytokine-cytokine receptor interaction negatively related to the ICB response was enriched in m6A cluster 2 (non-inflammatory phenotype) (Figure 4D). Therefore, we confirmed that m6A cluster 1 might represent an inflamed phenotype from the aspect of immunotherapy response.

### Developing m6A Scores and Correlating Them With Immune Phenotypes

**Figure 5:**
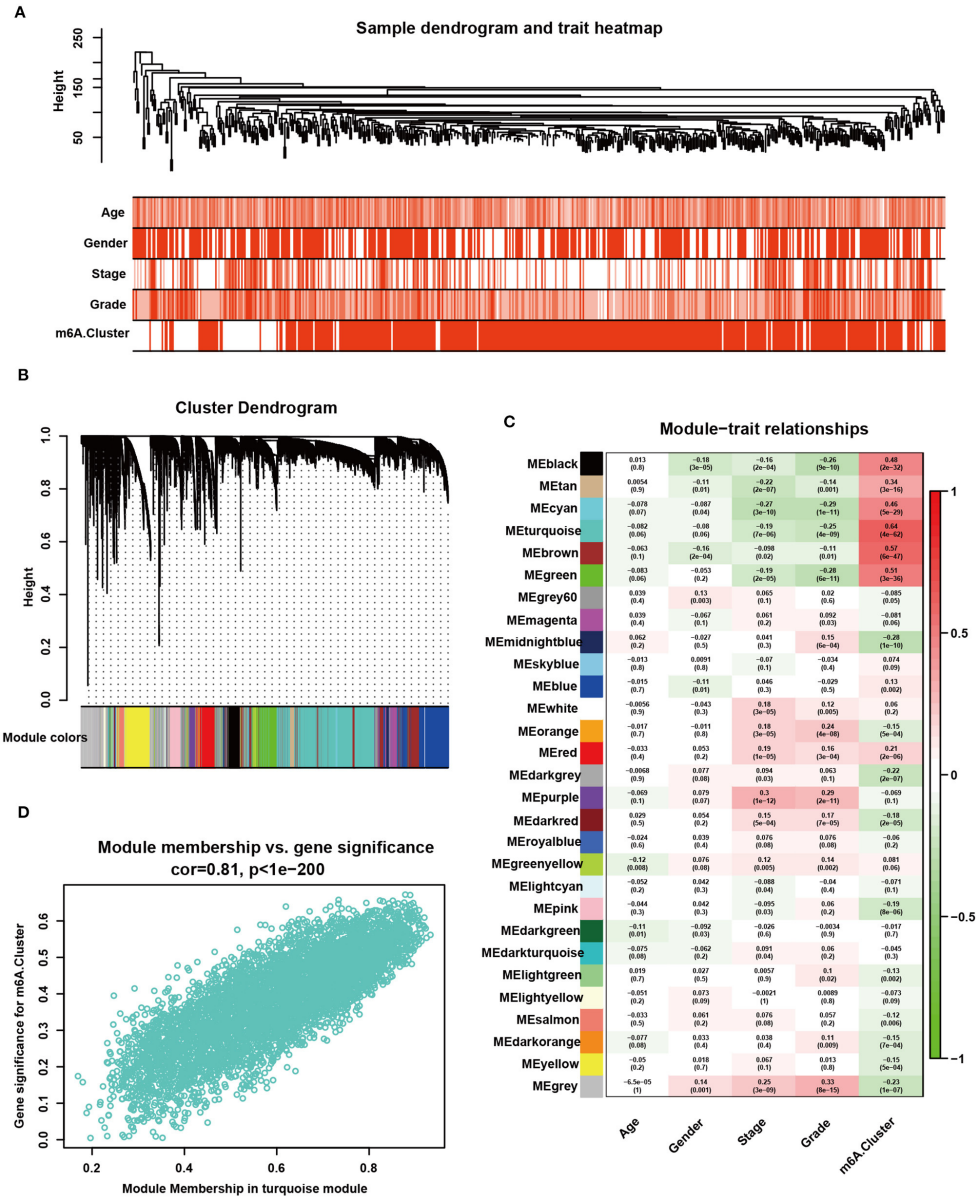
Detection and validation of m6A modification pattern-related modules by WGCNA. **(A)** Clustering dendrogram of 530 samples in the TCGA-KIRC dataset and heatmaps of clinical traits. The color intensity was related to older age, male sex, higher tumor stage, higher tumor grade, and m6A cluster 2. **(B)** Clustering dendrogram of differentially expressed genes. The dissimilarity was based on the topological overlap, and different modules were assigned to different colors. **(C)** Heatmap of the correlation between different gene modules and clinical characteristics. Red represents a positive correlation, and blue represents a negative correlation. **(D)** Scatter plot of membership in the turquoise module.

All tumor data from the TCGA-KIRC dataset were used to develop the gene co-expression network and to identify m6A cluster-related modules. All KIRC samples with full clinical characteristics were included in the co-expression analysis (Figure 5A). The “WGCNA” package was used to allocate genes with similar expression patterns into different modules. In this study, we chose the soft threshold as 7 (scale-free *R*^2^ = 0.85) to develop a scale-free network. As shown in Figure 5B, a total of 29 modules were recognized. The modules with the most significant association with clinical characteristics had the greatest biological meanings. The turquoise module was found to have the highest association with the m6A cluster (*r* = 0.64, *p* = 4e-64; Figure 5C). We chose the turquoise module to be analyzed in the subsequent steps, and the turquoise module was also related to tumor grade and stage. The genes in the turquoise modules were significantly co-expressed (cor = 0.81, *P* < 1e-200; Figure 5D). Among these genes, 2,214 were significantly related to prognosis (Supplementary Table 9). Then, the m6A score was calculated for individuals using the PCA algorithm.

**Figure 6:**
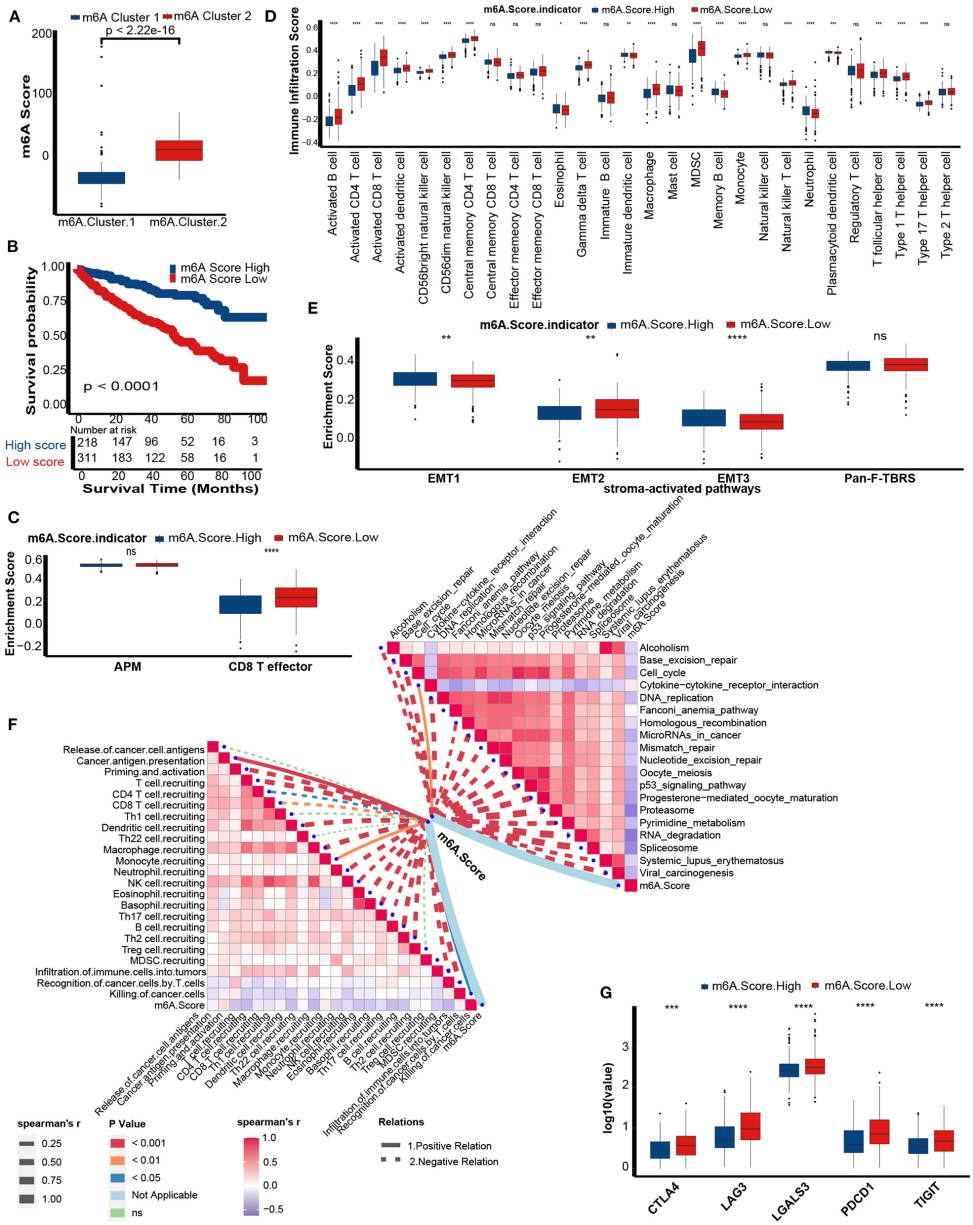
Differences in prognosis and immunological characteristics between the m6A score groups. **(A)** The m6A score in the two distinct m6A modification patterns. Kruskal-Wallis tests to calculate significant differences. **(B)** Survival analyses for the low (311 cases) and the high (218 cases) m6A score patient groups in the TCGA-KIRC cohort using Kaplan-Meier curves. m6A Score High, blue; m6A Score Low, red. **(C)** Activation of antigen processing machinery (APM) and CD8T effector pathways between the m6A Score group. M6A Score High, blue; m6A Score Low, red. **(D)** TME immune cell infiltration scores between the m6A score groups. M6A Score High, blue; m6A Score Low, red. **(E)** Activation of stroma-activated pathways in the m6A score group. M6A Score High, blue; m6A Score Low, red. **(F)** Spearman correlation analysis of m6A scores with activities of cancer immunity cycles (left) and immune-related pathways analyzed by ssGSEA (right). The thickness of the lines represents the relation strength. The different colors of the lines represent different *p*-values. The red bar plots represent a positive correlation, and the blue bar plots represent a negative correlation. **(G)** The histogram of immune checkpoint gene expression between the m6A score groups. M6A Score High, blue; m6A Score Low, red (ns, Not Significant; *P < 0.05; **P < 0.01; ***P < 0.001; ****P < 0.0001).

m6A score was lower in m6A cluster 1 (Figure 6A). Similar to the performance of m6A cluster 1, patients in the low m6A score group exhibited poorer prognosis than patients in the high m6A score group (Figure 6B). Also, m6A score still remained an independent prognosis factor in multivariate Cox regression analysis (*p* = 0.01, Supplementary Figure 3A). The Q-Q plot of the model showed that the residuals are approximately normally distributed (Supplementary Figure 3B) and the AUC at 5 years showed that the predictive accuracy of m6a score was comparative to tumor stage (Supplementary Figure 3C). There were consistent correlations between the m6A score and the immune phenotype. The CD8 T effector signatures were enriched in the low m6A score group (Figure 6C). The abundance of antitumor immune cells, including activated CD8 T cells, activated CD4 T cells, activated dendritic cells, CD56bright natural killer cells, central memory CD4 T cells, natural killer T cells, type 1 T helper cells, and type 17 T helper cells was significantly upregulated in the low m6A score group (Figure 6D). However, the abundance of protumor immune cells, including immature dendritic cells and plasmacytoid dendritic cells, was downregulated in the low m6A score group (Figure 6D). We validated the infiltration level of TIICs using Cibersort-ABS, xCell, and TIMER algorithm (Supplementary Figures 4–6). Generally, most of the algorithms showed that m6A score was negatively correlated with anti-tumor immune cells, including CD8 T cells, CD4 T cells, and natural killer T cell. Except TIMER algorithm showed that CD8 T cells was positively correlated with m6A score. This could be the calculation errors caused by different algorithms and mark gene sets. In addition, the EMT1 and EMT3 pathways were enriched in the high m6A score group (Figure 6E). Meanwhile, the m6A score was negatively related to the activities of several critical anticancer immunity cycles, such as priming and activation, T cell recruiting, CD8 T cell recruiting, CD4 T cell recruiting, dendritic cell recruiting, Th17 cell recruiting, and infiltration of immune cells into tumors (Figure 6F, Supplementary Table 10). These findings suggested that the low m6A score group may have an inflammatory phenotype.

As expected, m6A scores were negatively correlated with pathways that were positively related to the ICB response, such as RNA degradation, cell cycle, and DNA replication. In contrast, the m6A score was positively related to the cytokine-cytokine receptor interaction pathway, which was negatively related to the ICB response (Figure 6F, Supplementary Table 11). Finally, several common immune checkpoints, such as CTLA-4, PD-1, LAG-3, LAALS3, and TIGIT, were highly expressed in the low m6A score group (Figure 6G).

In summary, the m6A score predicts the immune phenotype and clinical response to ICB.

### Mutation Profiles of m6A Score Groups

**Figure 7:**
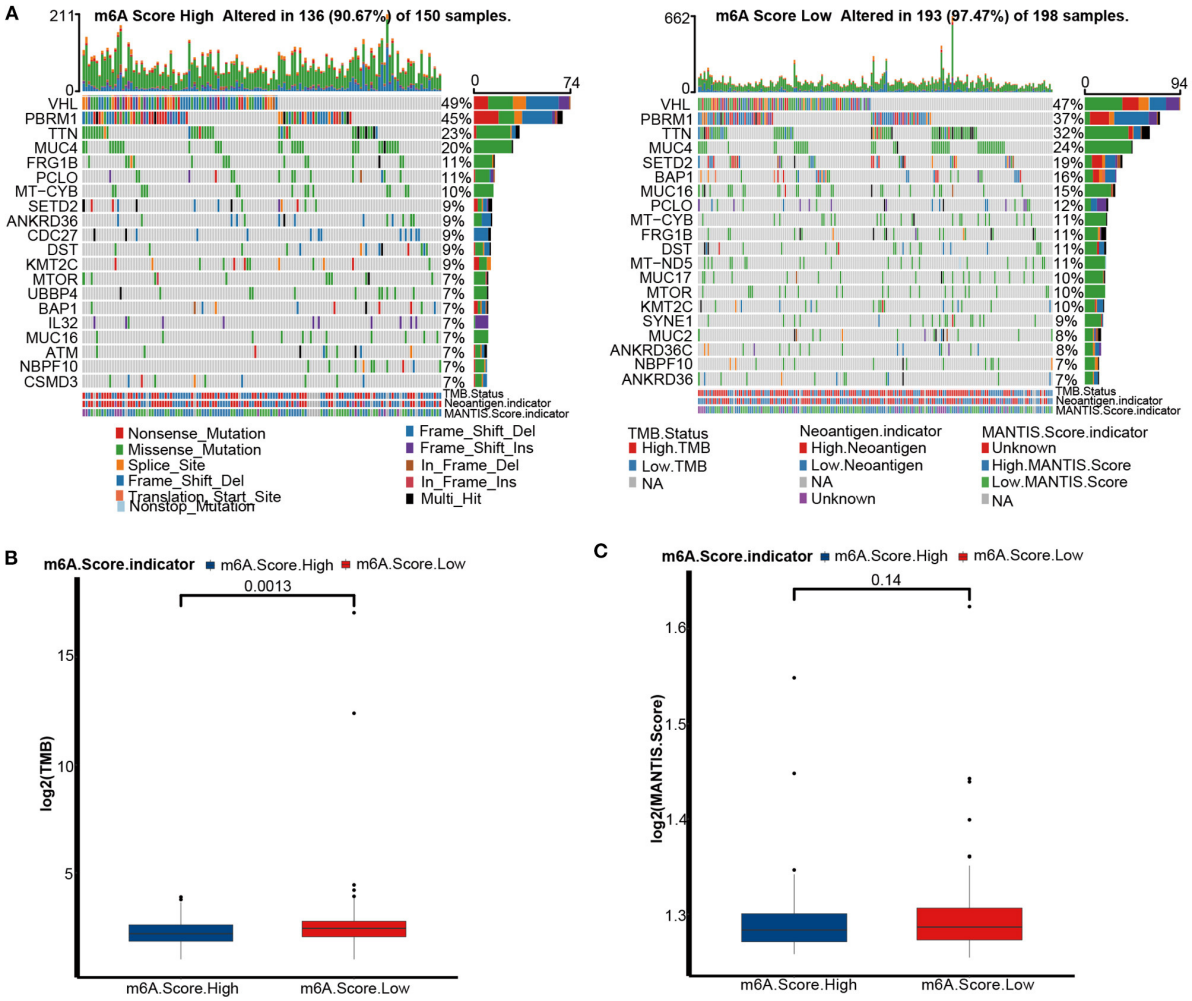
Tumor mutation burden (TMB) analyses of m6A score groups in the TCGA-KIRC cohort. **(A)** Mutation status in the high (left) and low (right) m6A score groups of the TCGA-KIRC dataset. Each column is related to individual patients. Upper bar plots represent TMB, right bar plots represent variant type proportions, and lower bar plots represent conversions or each sample. **(B)** The histogram of log2(TMB) between the m6A score groups. M6A Score High, blue; m6A Score Low, red. **(C)** The histogram of log2(MANTIS Score) between the m6A score groups. M6A Score High, blue; m6A Score Low, red.

Genomic mutations are a prominent factor in initiating malignancy. Here, we analyzed distribution differences in the top 20 somatic mutations between m6A score groups using the maftools R package. The most common mutations in KIRC were VHL and PBRM1. There was no difference in the VHL mutation between the m6A score groups (Figure 7A). The mutation frequencies of TTN (32 vs. 23%), SETD2 (19 vs. 9%), BAP1 (16 vs. 7%), and MUC16 (15 vs. 7%) were markedly higher in the low m6A score group suggesting that these mutations may be m6A score-specific mutations in KIRC. In general, a more extensive tumor mutation burden was presented in the low m6A score group than in the high m6A score group (97.4 vs. 90.67%) (Figure 7A). Consequently, the TMB quantification analysis revealed that the low m6A score group was markedly correlated with a higher TMB (Figure 7B). However, there was no difference in MSI status between the two m6A score groups (Figure 7C).

### External Validation of the m6A Score in GSE22541

**Figure 8:**
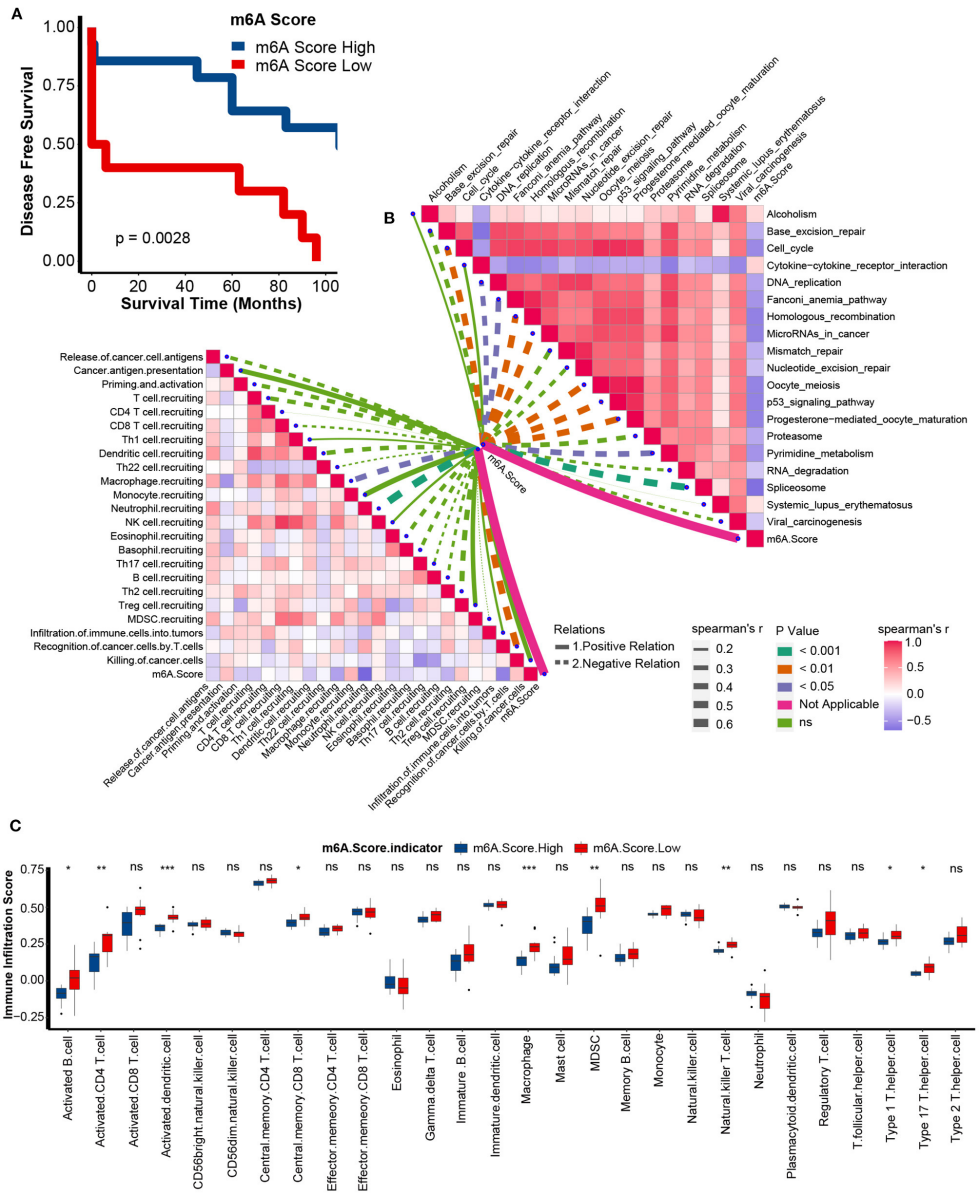
Validation of m6A score in the GSE22541 dataset. **(A)** Survival analyses for the low and high m6A score patient groups in the GSE22541 dataset using Kaplan-Meier curves. M6A Score High, blue; m6A Score Low, red. **(B)** Spearman correlation analysis of m6A scores with activities of cancer immunity cycles (left) and immune-related pathways analyzed by ssGSEA (right) in the GSE22541 dataset. The thickness of the lines represents the relation strength. The different colors of the lines represent different *p*-values. The red bar plots represent a positive correlation, and the blue bar plots represent a negative correlation. **(C)** TME immune cell infiltration scores between the m6A score groups in the GSE22541 dataset. M6A Score High, blue; m6A Score Low, red (ns, Not Significant; *P < 0.05; **P < 0.01; ***P < 0.001).

Similar to the performance of the m6A score in the TCGA-KIRC cohort, we found that the low m6A score group had a poorer prognosis in the GSE22541 cohort as well (Figure 8A). Meanwhile, the m6A score was negatively correlated with the activities of many anticancer immunity cycles, such as the recognition of cancer cells by T cells (Figure 8B, Supplementary Table 12). Furthermore, the infiltration levels of activated CD8 T cells, activated CD4 T cells, activated dendritic cells, central memory CD8 T cells, natural killer T cells, type 1 T helper cells, and type 17 T helper cells were significantly higher in the low m6A score group (Figure 8C). Finally, the m6A score was negatively related to most pathways that predicted higher ICB response rates (Figure 8B, Supplementary Table 13). These results confirmed that the m6A score might be a robust predictor of immune phenotype, prognosis, and ICB response.

### Role of the m6A Score in Predicting the Response to Targeted Therapy and Immunotherapy

**Figure 9:**
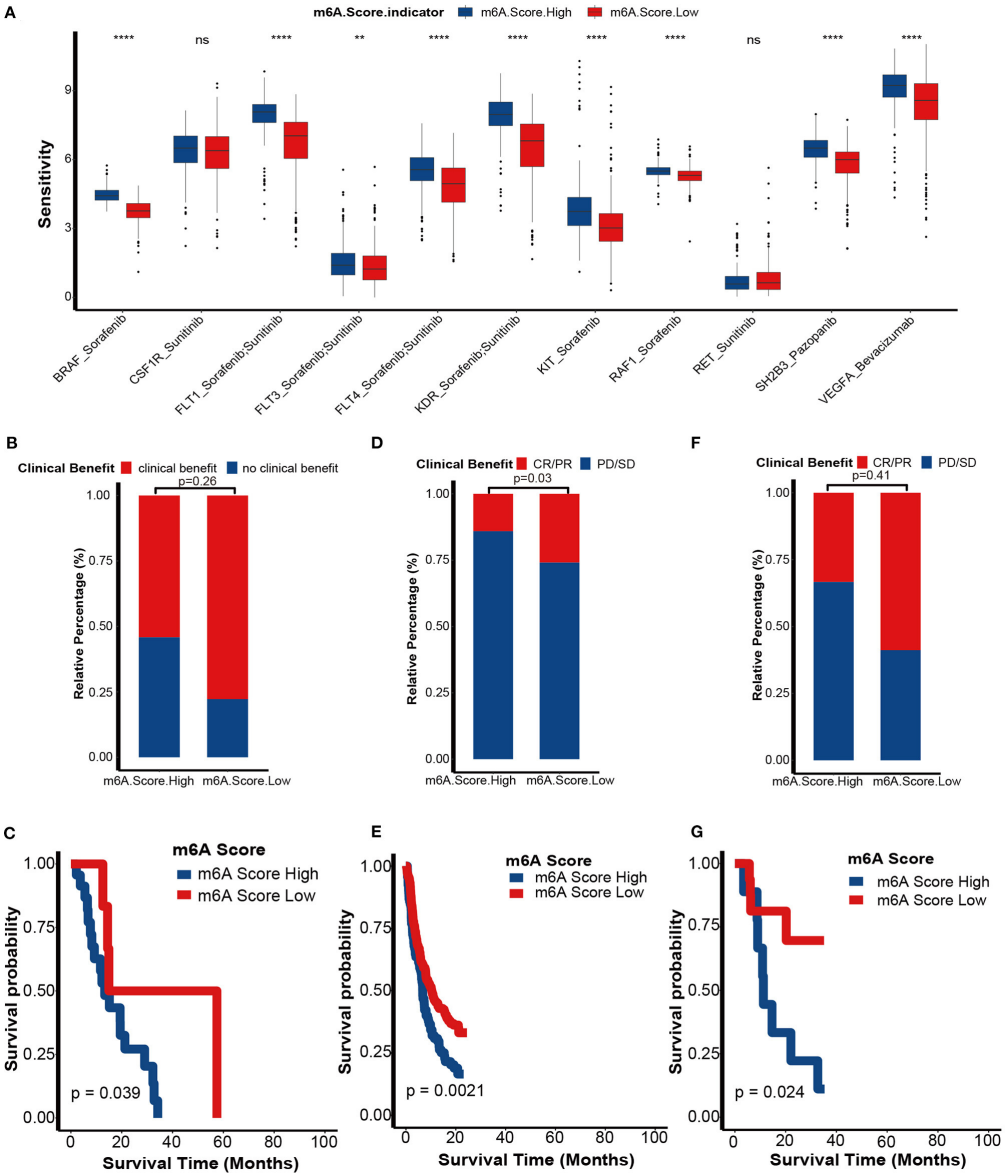
Role of m6A score in predicting sensitivities of targeted therapy and immunotherapy. **(A)** The differences in sensitivities of targeted therapy between m6A score groups by analyzing data from the DrugBank dataset. m6A Score High, blue; m6A Score Low, red. **(B)** Proportion of patients with clinical benefit to immunotherapy between the different m6A score groups in an RCC immunotherapy dataset (PMID29301960). **(C)** Survival analyses for the low and high m6A score patient groups in the RCCICI dataset using Kaplan-Meier curves. **(D)** Proportion of patients with clinical benefit to immunotherapy between the different m6A score groups in IMvigor210 dataset. **(E)** Survival analyses for the low and high m6A score patient groups in the IMvigor210 dataset using Kaplan-Meier curves. **(F)** Proportion of patients with clinical benefit to immunotherapy between the different m6A score groups in the GSE78220 dataset. **(G)** Survival analyses for the low and high m6A score patient groups in the GSE78220 dataset using Kaplan-Meier curves (CR, complete response; PR, partial response; SD, stable disease; PD, progressive disease; ns, Not Significant; **P < 0.01; ****P < 0.0001).

We further explored the role of the m6A score in guiding clinical decision making in KIRC. First, we found that the sensitivities of many anticancer drugs were significantly different between m6A score groups (Supplementary Table 14). Targeted therapy was the first-line treatment option for advanced KIRC. Here, we collected the targeted therapy drugs used in KIRC and their targeted genes from the DrugBank database: sorafenib with its targeted genes including BRAF, FLT1, FLT3, FLT4, KDR, KIT, and RAF1; sunitinib with its targeted genes including CSF1R, FLT1, FLT3, FLT4, KDR, and RET; pazopanib with its targeted gene SH2B3; and bevacizumab with its targeted gene VEGFA. Interestingly, all targeted therapy drug sensitivities were significantly lower in the low m6A score group (Figure 9A). These results indicate that the m6A score may identify suitable candidates to receive targeted therapy.

Although findings from TCGA-KIRC and GSE22541 cohorts suggested that the m6A score predicts ICB response, it would be more convincing to validate these results in cohorts that received ICB. First, in a KIRC cohort that received anti-PD-1 therapy (nivolumab), we demonstrated that the clinical benefit rate was higher in the low m6A score group than in the high m6A score group (*p* = 0.26; Figure 9B). Regrettably, because of the small sample size, we didn't find significantly differences. The prognosis of the low m6A score group was better than in the high m6A score group (*p* = 0.039; Figure 9C). It is worth noting that this survival outcome was contrary to the results showing that the prognosis of the low m6A score group was worse in the TCGA-KIRC and GSE22541 cohorts. These differences in outcome were due to the response rate of immunotherapy being more likely to determine the prognosis of an immunotherapy cohort when compared to other prognostic risk factors, such as the m6A score. Additionally, we successfully validated the role of the m6A score in predicting the response to ICB in two other cancer cohorts, including the IMvigor210 cohort (bladder cancer) and GSE78220 cohort (melanoma) (Figures 9D–G). These findings revealed that this m6A score may represent a generalized predictor for response to ICB in other cancer types as well.

## Discussion

m6A modification plays a critical role in regulating the immune status of the TME in various cancers (10). However, the role of m6A in modifying immune characteristics in KIRC needs to be further explored. In this manuscript, we identified two independent m6A modification patterns with distinct biological functions, immunological characteristics, and prognoses. Then, we developed an m6A score algorithm to quantify an individual's m6A modification pattern, which was independently validated in external cohorts.

There are some studies reporting the function of m6A modification in the progression, prognosis and the TME in KIRC, indicating the potential key role of m6A regulators in KIRC. Strick et al. reported that ALKBH5 and FTO were significantly downregulated in KIRC compared to normal tissues, and their low expression predicted poorer prognosis (37). However, Zhang et al. found that ALKBH5 was highly expressed in KIRC compared to normal tissues, and high expression of ALKBH5 promoted progression of KIRC (38). Notably, a simple analysis of a single m6A gene in KIRC may lead to obvious contradictory results. These differences in results might be because m6A modification is an extremely complex process that is regulated by writers, erasers, and readers. Therefore, systematic analysis of all m6A genes may more comprehensively reflect the m6A modification pattern in the TME. To date, there are some studies performing systematic analysis of multiple m6A genes using bioinformatics algorithms and reported that the m6A modification pattern predicted progression and prognosis of KIRC. Chen et al. systematically analyzed the global m6A modification pattern in KIRC and correlated it with cancer-related gene expressions (39). Zhou et al. found a close relationship between genetic alterations of m6A regulators with clinical characteristics in KIRC (40). Zhang et al. (41), Wang et al. (42), and Chen et al. (43) systematically analyzed the m6A regulators in KIRC and developed a METTL3 and METTL14 based risk score for the prognosis of KIRC. Zhao et al. developed a risk score based on three m6A regulators, including METTL3, METTL14, and HNRNPA2B1 (44). However, all of them have not correlated m6A regulators with TME. Fang et al. systematically analyzed 16 m6A regulators and correlated them with TME. Also, they developed a four-m6A-regulators based risk score only for the prognosis (45). But they have not analyzed the relationship between m6A regulators and ICB response. In addition, their risk score can't predict the immune phenotypes of KIRC and quantify the m6A modification pattern of an individual patient.

Abnormal m6A modification patterns promote the development of cancers (8). In our study, we found that the expression profiles between m6A writers and m6A erasers were imbalanced. Theoretically, these imbalanced expression profiles may cause abnormal m6A modification patterns and consequently lead to KIRC development. In addition, the majority of m6A genes were related to prognosis. More importantly, these m6A genes were related to each other and formed a close interaction network. These findings prompted us to perform a comprehensive clustering analysis instead of analyzing the role of a single m6A gene.

Zhang et al. identified three different m6A clusters in gastric cancer based on 21 m6A genes. After analyzing the landscapes of immunological characteristics, prognosis, and other functions, they connected the three m6A clusters to different immune phenotypes, including inflammatory, excluded, and deserted phenotypes (16). Indeed, the excluded and deserted phenotypes can be unified into a non-inflammatory phenotype. In our study, we similarly identified two m6A clusters that reflected different immune phenotypes.

The TME is a complex system composed of cancer cells, various TIICs, and an extracellular matrix. These TIICs play a distinct role in regulating anticancer immunity. In general, CD8 T cells and natural killer cells were the most important cytotoxic cells that killed tumor cells. Other antitumor TIICs included CD4 T cells, type 1 T helper cells, and type 17 T helper cells (46). Regulatory T cells are recognized as the most important protumor TIICs (46). In addition, there are various immunomodulators, including chemokines, MHC, immune stimulators, immune inhibitors, and receptors (28). The comprehensive effects of these different TIICs and immunomodulators determine the direction of the anticancer immune response. The activities of the anticancer immune response determine the fate of cancer cells. In this study, T cell recruitment activity was higher in m6A cluster 1. Consequently, activated CD8 T cells, activated CD4 T cells, natural killer cells, and type 17 T helper cells were enriched in m6A cluster 1. In contrast, regulatory T cells were enriched in m6A cluster 2. Stromal pathways, such as EMT and Pan-FTBRS signatures, may inhibit anticancer immunity (20). Consistently, the enrichment score of these immune-inhibiting pathways was lower in m6A cluster 1. This evidence indicates that the m6A cluster 1 belongs to an inflammatory phenotype, while m6A cluster 2 reflected a non-inflammatory phenotype. Additionally, pathways that were positively related to the ICB response were enriched in the m6A cluster 1. Therefore, m6A cluster 1 was theoretically more sensitive to ICB.

We developed the m6A score using WGCNA and PCA algorithms. The WGCNA algorithm identified gene sets that are highly related to the specific biological behavior and clinical phenotype of a cohort (32). Genes in these sets are highly correlated with each other. Based on this, the PCA algorithm further calculated the score of genes with the highest correlation with the m6A cluster, while decreasing the contributions from other factors (16, 34). As a result, the m6A score accurately reflected the m6A clusters. In our study, the low m6A score group indicated m6A cluster 1, while the high m6A score group indicated m6A cluster 2. We then evaluated the value of the m6A score in predicting immune phenotypes, prognosis, and ICB response. In general, the m6A score was negatively related to anticancer immunity in the TME. Therefore, the low m6A score group indicated an inflamed phenotype. As a result, the m6A score was negatively related to pathways that were positively related to ICB response.

Theoretically, patients with an inflammatory phenotype may have a better prognosis. However, we found that patient prognosis in the low m6A score group was worse, even though the low m6A score group had an inflammatory phenotype. This could be because several critical inhibitor immune checkpoints, including CTLA-4, PD-1, LAG-3, LAALS3, and TIGIT, were significantly highly expressed in the low m6A score group. Higher expression of these immune checkpoints may limit cytotoxic immune cell activities in the TME, such as CD8 T cells, causing these cytotoxic cells to be in an exhausted functional state (47, 48). Finally, the robustness of the m6A score was invalidated in external cohorts.

Both targeted therapy and ICB have been recommended as first-line treatments for advanced KIRC (2–4). However, it is difficult to determine an individual's optimal treatment option, which prompted us to explore more accurate predictive biomarkers. Here, the m6A score may be a potential biomarker to guide clinical decision-making and help us achieve individualized and precision treatment. First, we identified a highly consistent result that all targeted therapy drugs' sensitivities were significantly lower in the low m6A score group, indicating that patients with high m6A scores might be suitable candidates to receive targeted therapy. In contrast, patients with low m6A scores may be the optimal candidates to receive ICB. More importantly, we demonstrated that this m6A score may be a generalized predictor for the response to ICB in other cancer types.

Several inevitable shortcomings exist in this study. First, all conclusions came from public databases, including validations. This weakens the use of these conclusions for the future. Therefore, it is necessary to validate these findings with experiments *in vivo* and *in vitro* and more data from our center in the future. Second, in order to enlarge our sample size and verify our results, we pooled data from TCGA and GEO together. However, despite the inevitable analysis error caused by different sequencing platforms, we found that the results found in TCGA can be successfully verified in multiple independent external datasets, which enhanced the reliability of our results. Third, it is difficult to unify the same cutoff value of the m6A score in different cohorts due to the differences in sequencing platform and batch effects. Alternatively, we used the survcutpoint function to calculate the optimal cutoff values.

In conclusion, this work revealed that m6A modification patterns played significant role in regulating the TME of KIRC, including immunological characters, mutation profiles and other functional pathways. Based on the comprehensive m6A patterns, we first identified m6A clusters and m6A scores to elucidate immune phenotypes and to predict the prognosis and immunotherapy response in KIRC. Finally, the m6A clusters and m6A scores can guide urologists for making more precise clinical decision.

## Data Availability Statement

Publicly available datasets were analyzed in this study. This data can be found at: TCGA-KIRC, https://portal.gdc.cancer.gov/; The copy number variation (CNV) data, http://xena.ucsc.edu/; The microsatellite instability (MSI) data, https://pubmed.ncbi.nlm.nih.gov/29850653/; GSE22541, GSE78220, https://www.ncbi.nlm.nih.gov/geo/; PMID29301960, https://pubmed.ncbi.nlm.nih.gov/29301960/; IMvigor210, http://research-pub.gene.com/IMvigor210CoreBiologies/.

## Author Contributions

HL and JH performed analyses and drafted the manuscript. AY, BO, and TG searched and downloaded the original datasets from TCGA and GEO. HL, JH, and JL contributed to statistical analyses. JL and CC collected and assembled clinical data. HL, TG, and JC edited the pictures. XZ and JC conceived and supervised the study. All authors contributed to writing the manuscript and reviewed and approved the final manuscript.

## Conflict of Interest

The authors declare that the research was conducted in the absence of any commercial or financial relationships that could be construed as a potential conflict of interest.

## Supplementary Material

Supplementary Figure 1 The workflow of the study design.

Supplementary Figure 2 GO and KEGG analysis of differentially expressed genes between m6A cluster 1 and 2. **(A)** Biological process. **(B)** Cellular component. **(C)** Molecular function. **(D)** KEGG analysis.

Supplementary Figure 3 Prognosis value of m6A score in multivariate regression model. **(A)** Forest plot of age, gender, tumor grade, tumor stage, and m6A score in multivariate regression model; **(B)** Q-Q plot of the multivariate regression model; **(C)** The five-year predictive value of m6A score, tumor stage, tumor grade, and age in TCGA-KIRC.

Supplementary Figure 4 Correlations between m6A score and tumor-infiltrating immune cells calculated with Cibersort-ABS algorithm.

Supplementary Figure 5 Correlations between m6A score and tumor-infiltrating immune cells calculated with xCell algorithm.

Supplementary Figure 6 Correlations between m6A score and tumor-infiltrating immune cells calculated with TIMER algorithm.

Supplementary Table 1 Information for datasets selected in this study.

Supplementary Table 2 The gene sets for TME cell infiltration enrichment analysis.

Supplementary Table 3 The signatures related to response of immunotherapy.

Supplementary Table 4 Spearman correlation values for 24 regulators in renal cell carcinoma.

Supplementary Table 5 Differentially expressed genes between m6A cluster 1 and 2.

Supplementary Table 6 Functional annotation of differentially expressed genes between m6a cluster 1 and 2 (GO).

Supplementary Table 7 Functional annotation of differentially expressed genes between m6a cluster 1 and 2 (KEGG).

Supplementary Table 8 Differences in HALLMARK pathway GSVA enrichment between m6A Cluster 1 and 2.

Supplementary Table 9 Prognosis-related genes from WGCNA between m6A cluster 1 and 2.

Supplementary Table 10 Correlations between m6A score and anticancer immunity.

Supplementary Table 11 Correlations between m6A score and immunotherapy associated signatures.

Supplementary Table 12 Correlations between m6A score and anticancer immunity in GSE22541.

Supplementary Table 13 Correlations between m6A Score and immunotherapy associated signatures in GSE22541.

Supplementary Table 14 Differences in the expression of target genes of antitumor drugs between high and low m6A score groups.
