# Supplementary material for: RNA Modification of N6-Methyladenosine Predicts Immune Phenotypes and Therapeutic Opportunities in Kidney Renal Clear Cell Carcinoma
Source: Front Oncol. 2021 Mar 18;11:642159. doi: 10.3389/fonc.2021.642159 (PMC8013979; doi:10.3389/fonc.2021.642159)
Supplement: Supplementary Figure 1 — The workflow of the study design. [file Image_1.pdf]

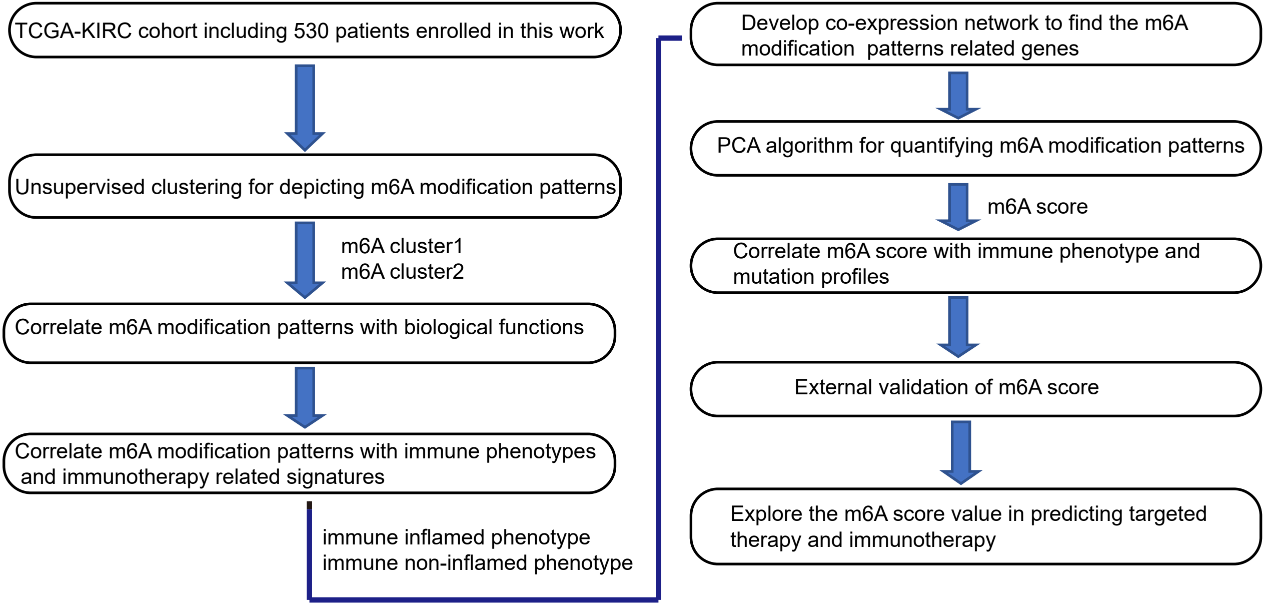
TCGA-KIRC cohort including 530 patients enrolled in this work
Unsupervised clustering for depicting m6A modification patterns
m6A cluster1
m6A cluster2
Correlate m6A modification patterns with biological functions
Correlate m6A modification patterns with immune phenotypes and immunotherapy related signatures
immune inflamed phenotype
immune non-inflamed phenotype
Develop co-expression network to find the m6A modification patterns related genes
PCA algorithm for quantifying m6A modification patterns
m6A score
Correlate m6A score with immune phenotype and mutation profiles
External validation of m6A score
Explore the m6A score value in predicting targeted therapy and immunotherapy
