# Supplementary figures and images for: RNA Modification of N6-Methyladenosine Predicts Immune Phenotypes and Therapeutic Opportunities in Kidney Renal Clear Cell Carcinoma
Source: Front Oncol. 2021 Mar 18;11:642159. doi: 10.3389/fonc.2021.642159 (PMC8013979; doi:10.3389/fonc.2021.642159)

A

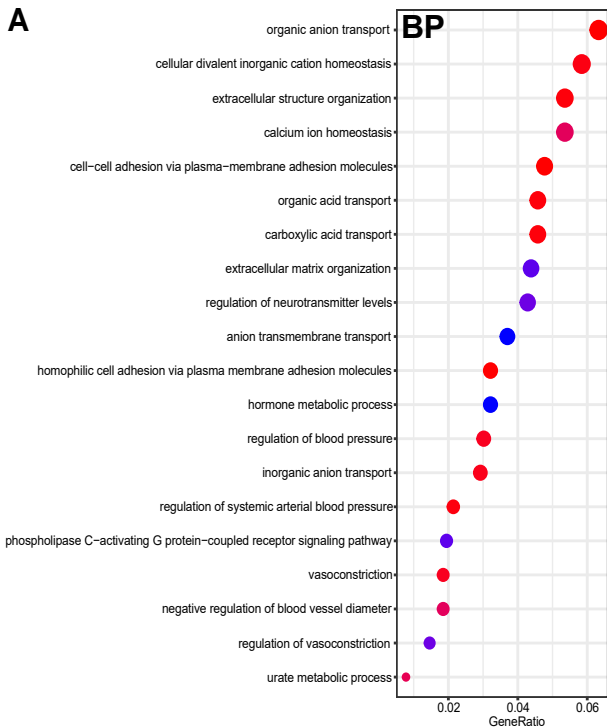

B

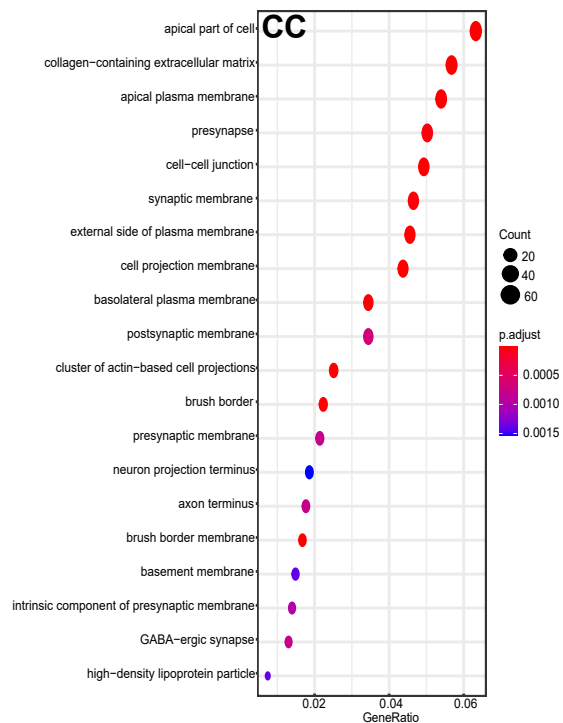

C

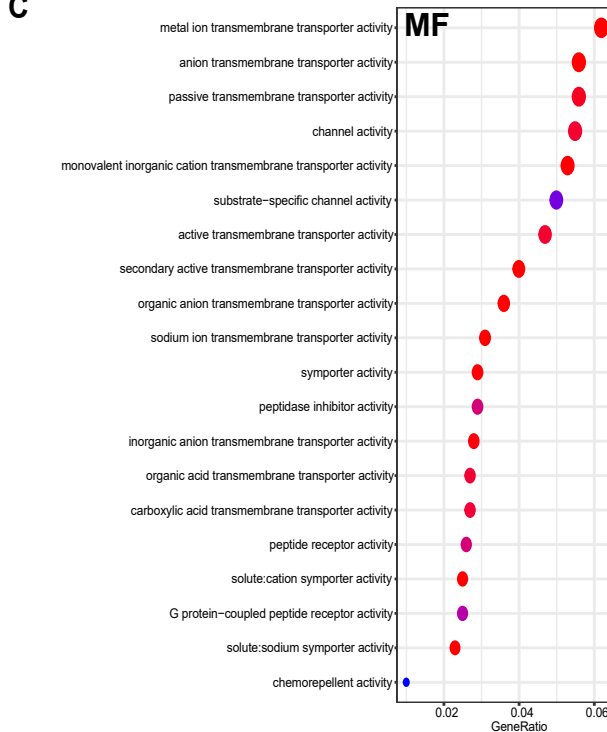

D

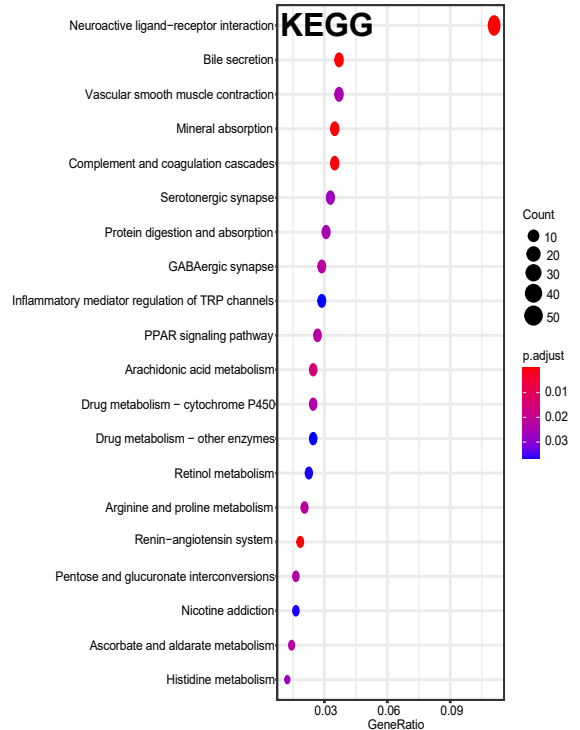

Supplement: Supplementary Figure 2 — GO and KEGG analysis of differentially expressed genes between m6A cluster 1 and 2. (A) Biological process. (B) Cellular component. (C) Molecular function. (D) KEGG analysis. [file Image_2.pdf]

**A**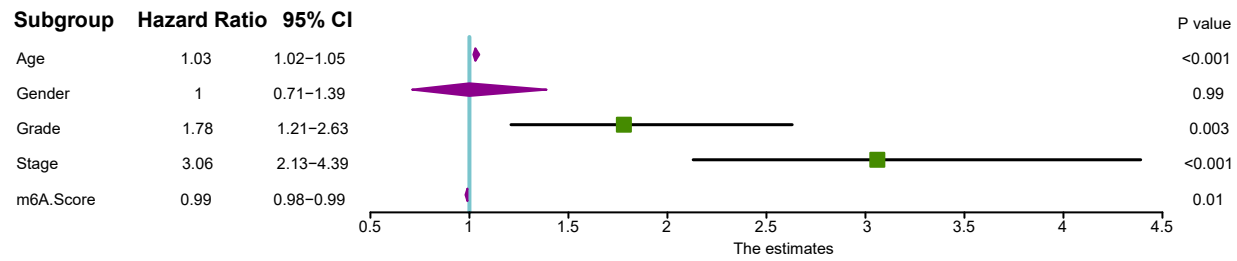**B**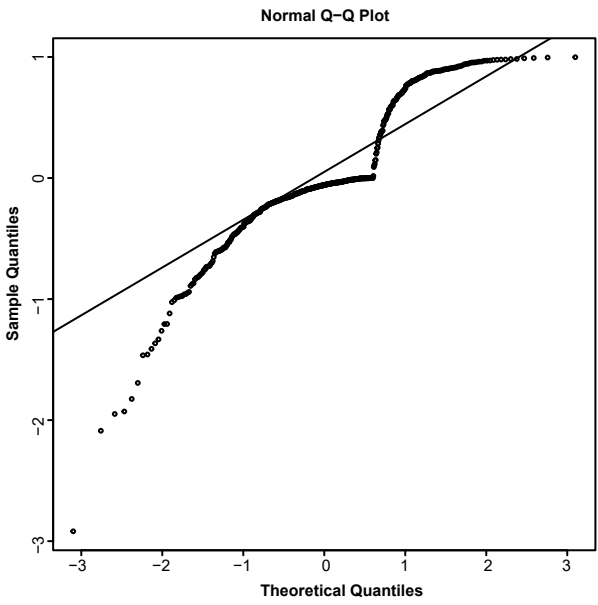**C**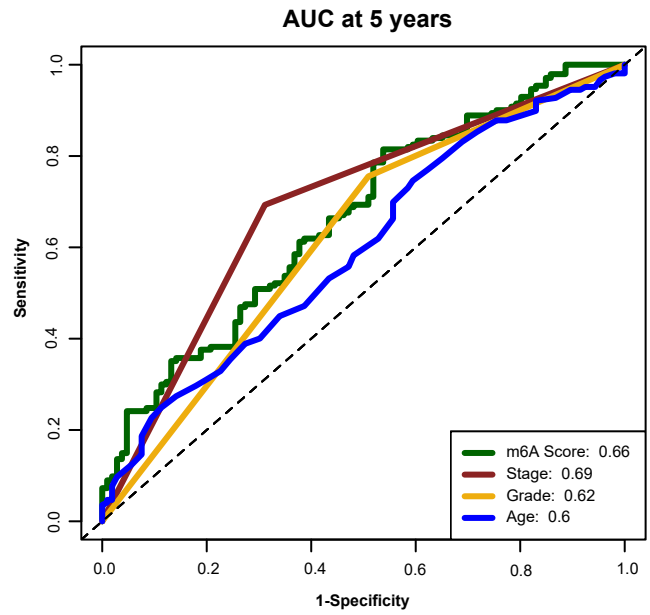

Supplement: Supplementary Figure 3 — Prognosis value of m6A score in multivariate regression model. (A) Forest plot of age, gender, tumor grade, tumor stage, and m6A score in multivariate regression model; (B) Q-Q plot of the multivariate regression model; (C) The five-year predictive value of m6A score, tumor stage, tumor grade, and age in TCGA-KIRC. [file Image_3.pdf]

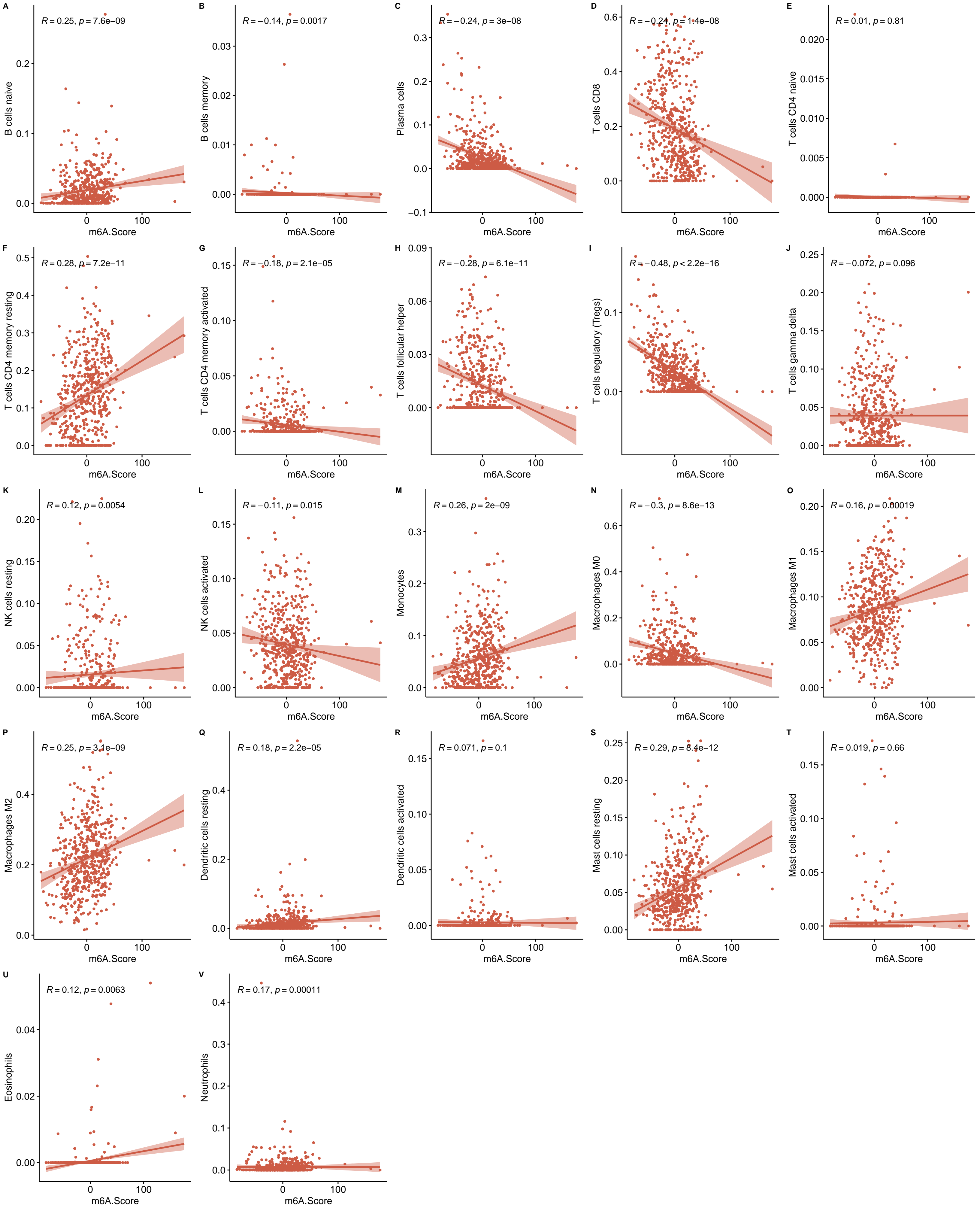

Supplement: Supplementary Figure 4 — Correlations between m6A score and tumor-infiltrating immune cells calculated with Cibersort-ABS algorithm. [file Image_4.pdf]

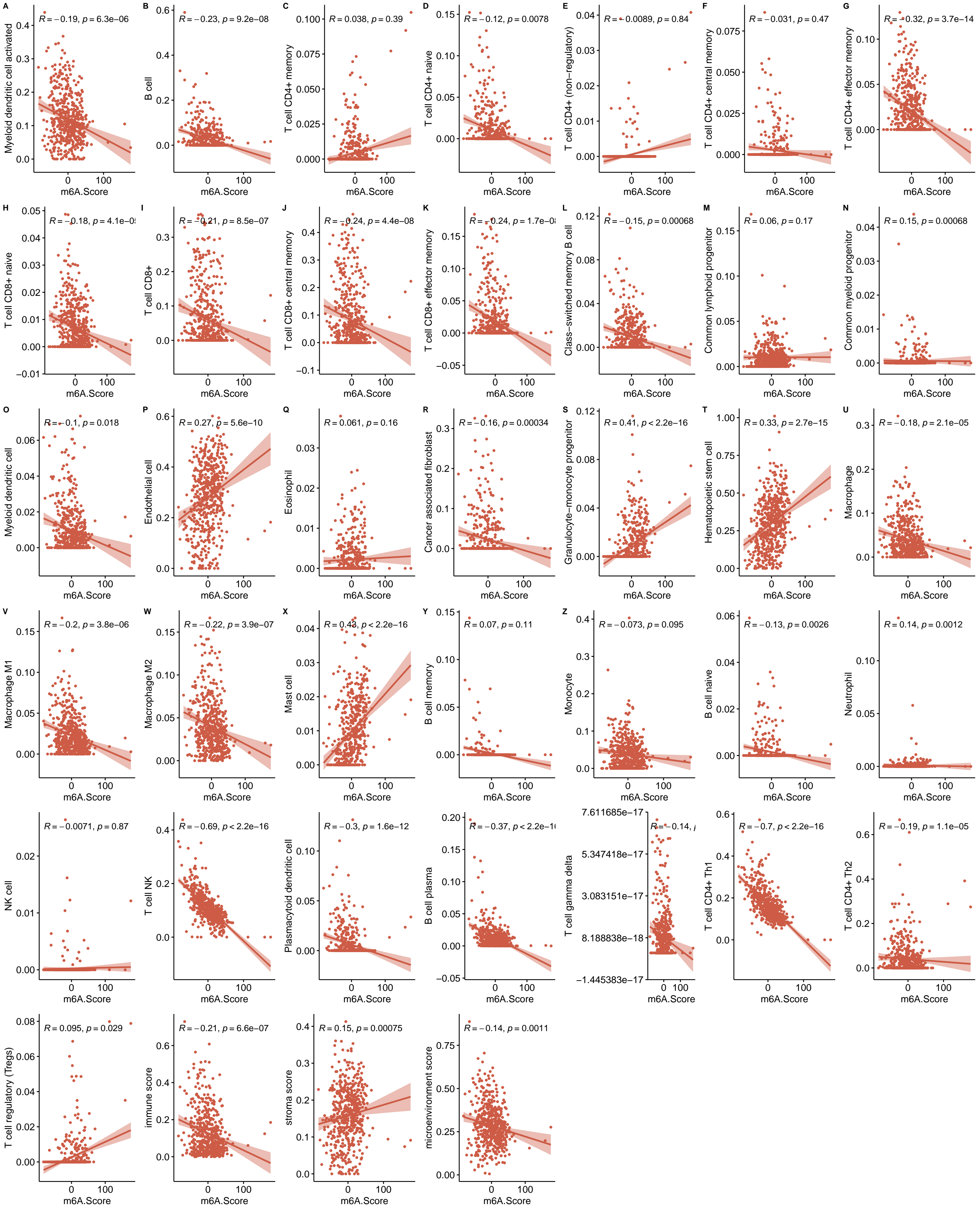

Supplement: Supplementary Figure 5 — Correlations between m6A score and tumor-infiltrating immune cells calculated with xCell algorithm. [file Image_5.pdf]

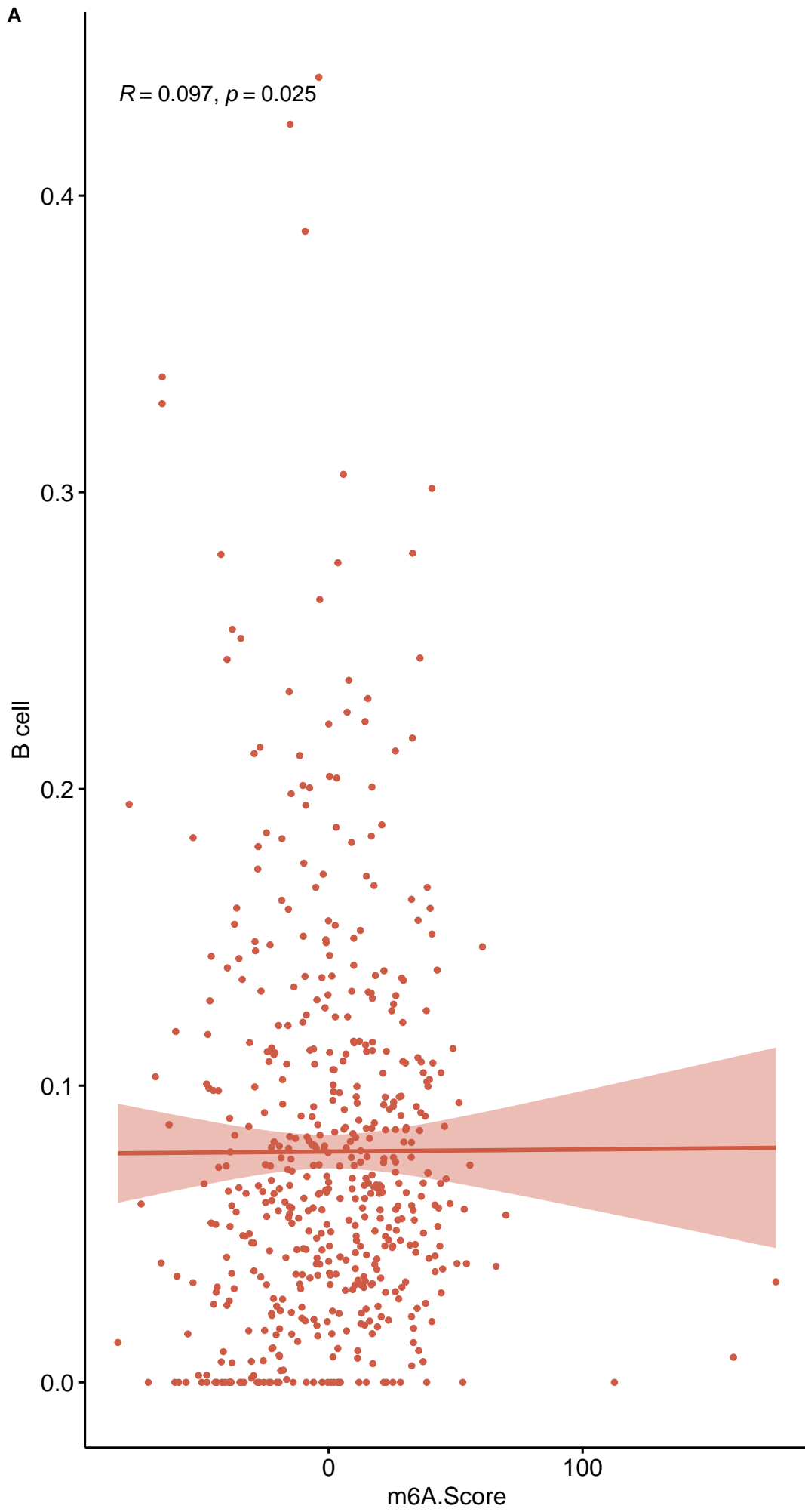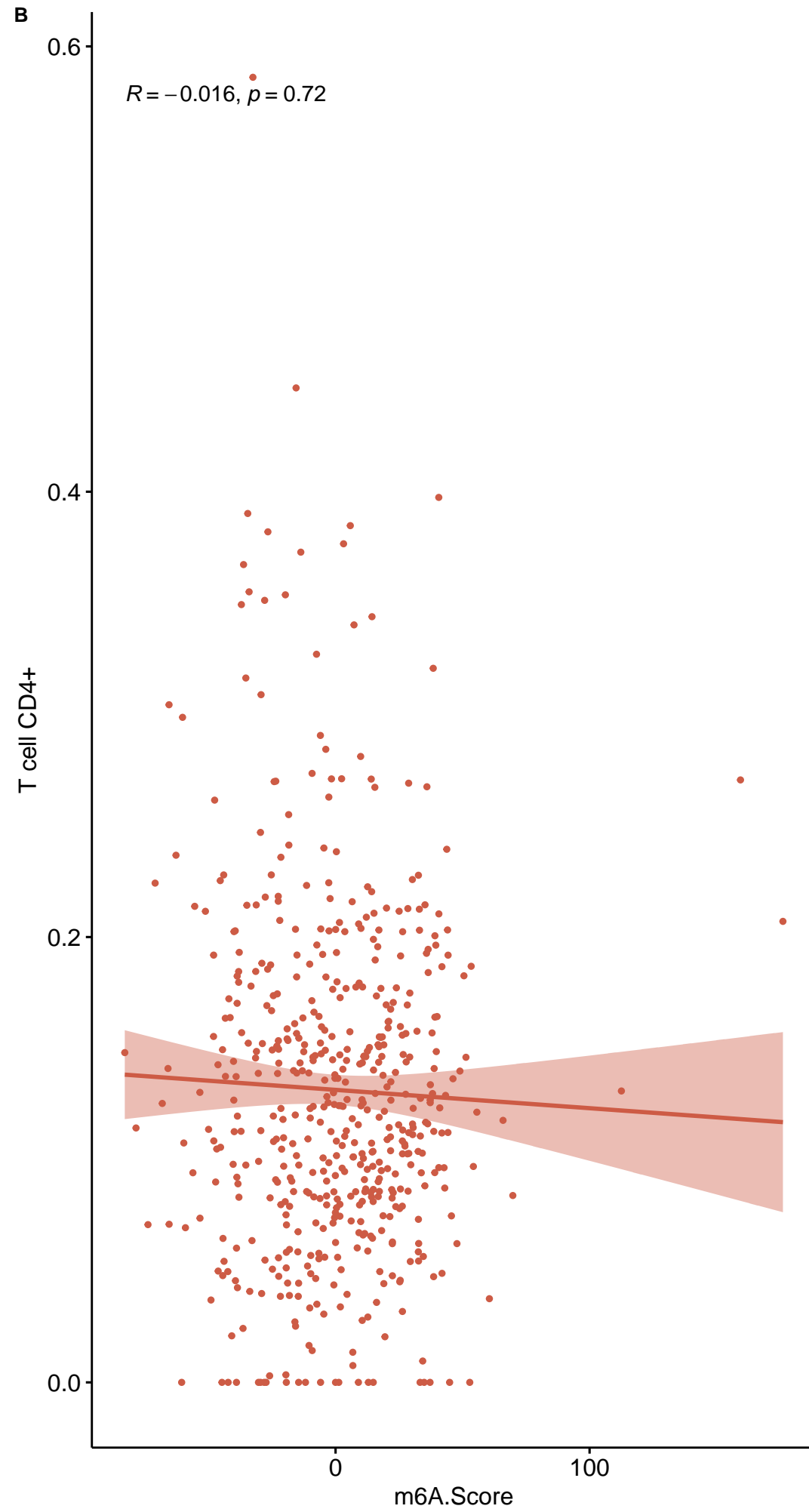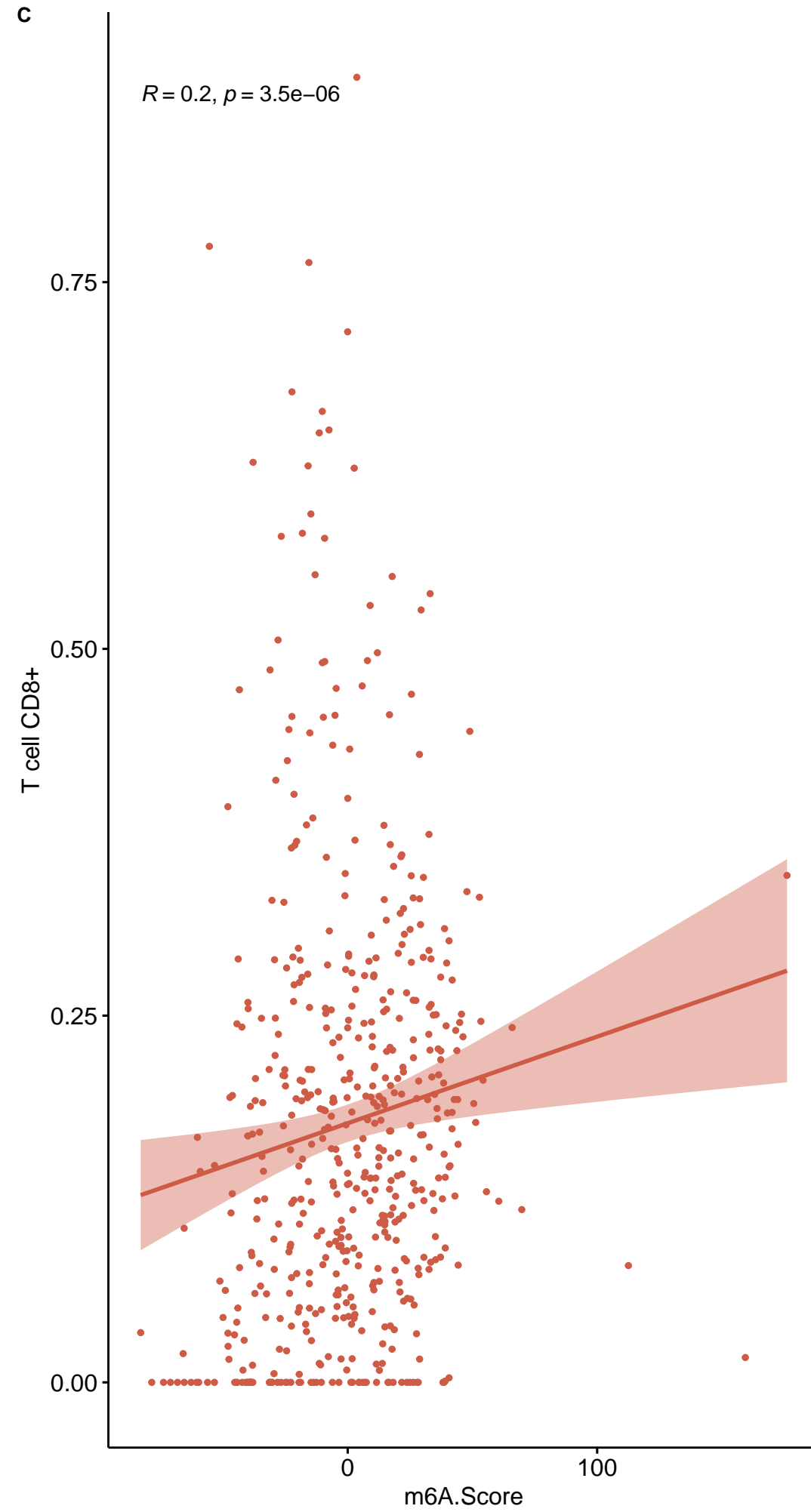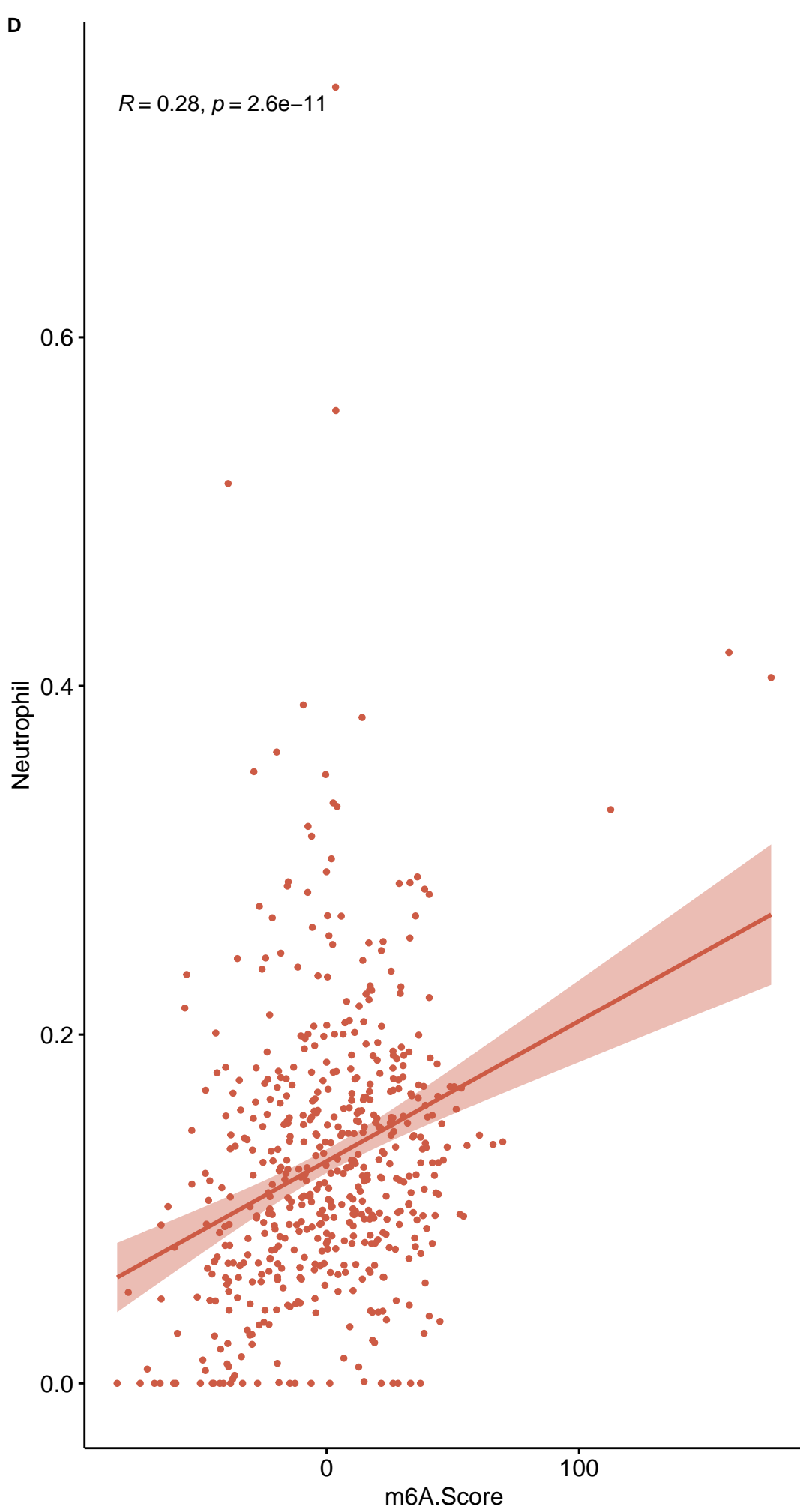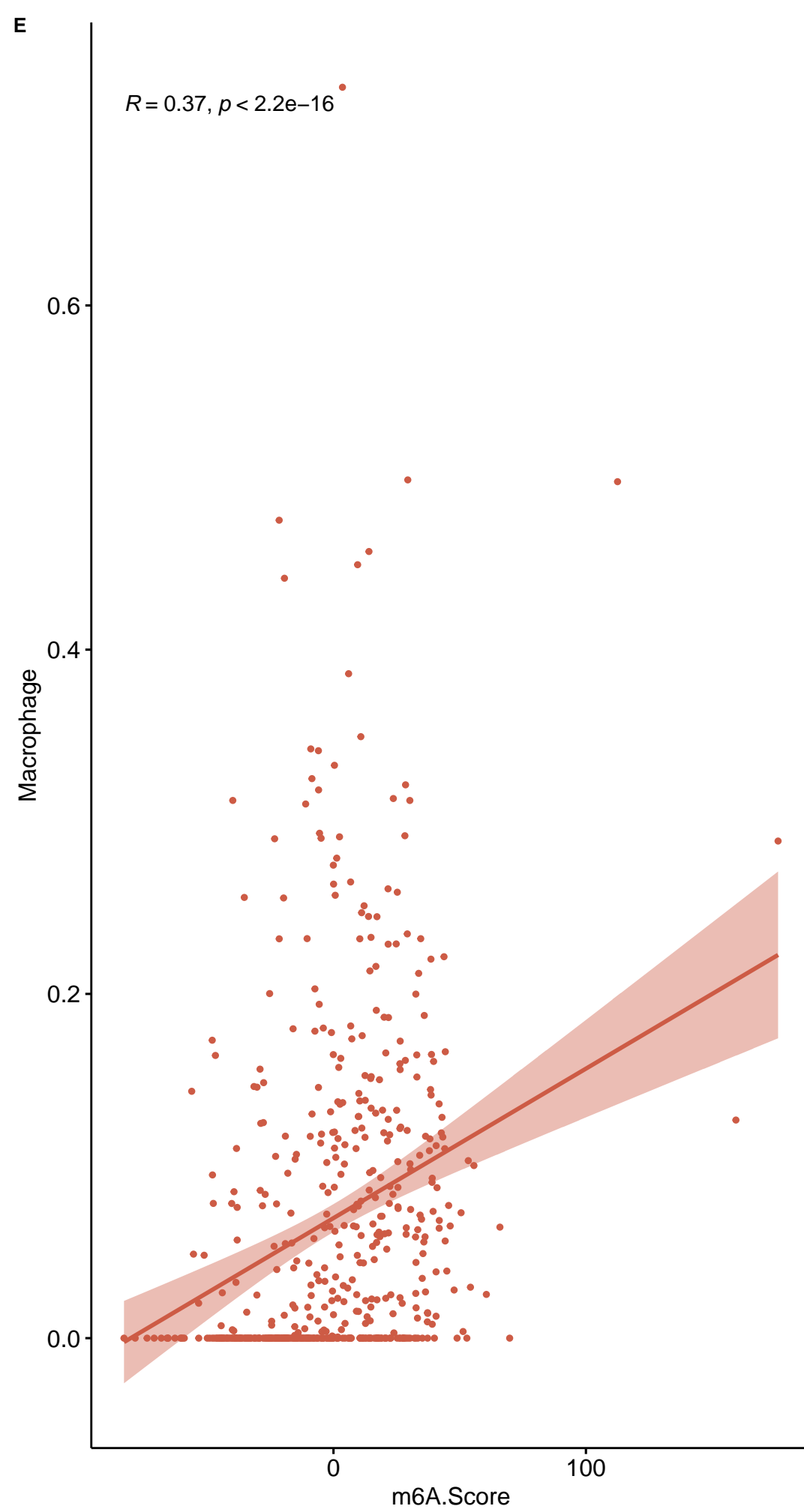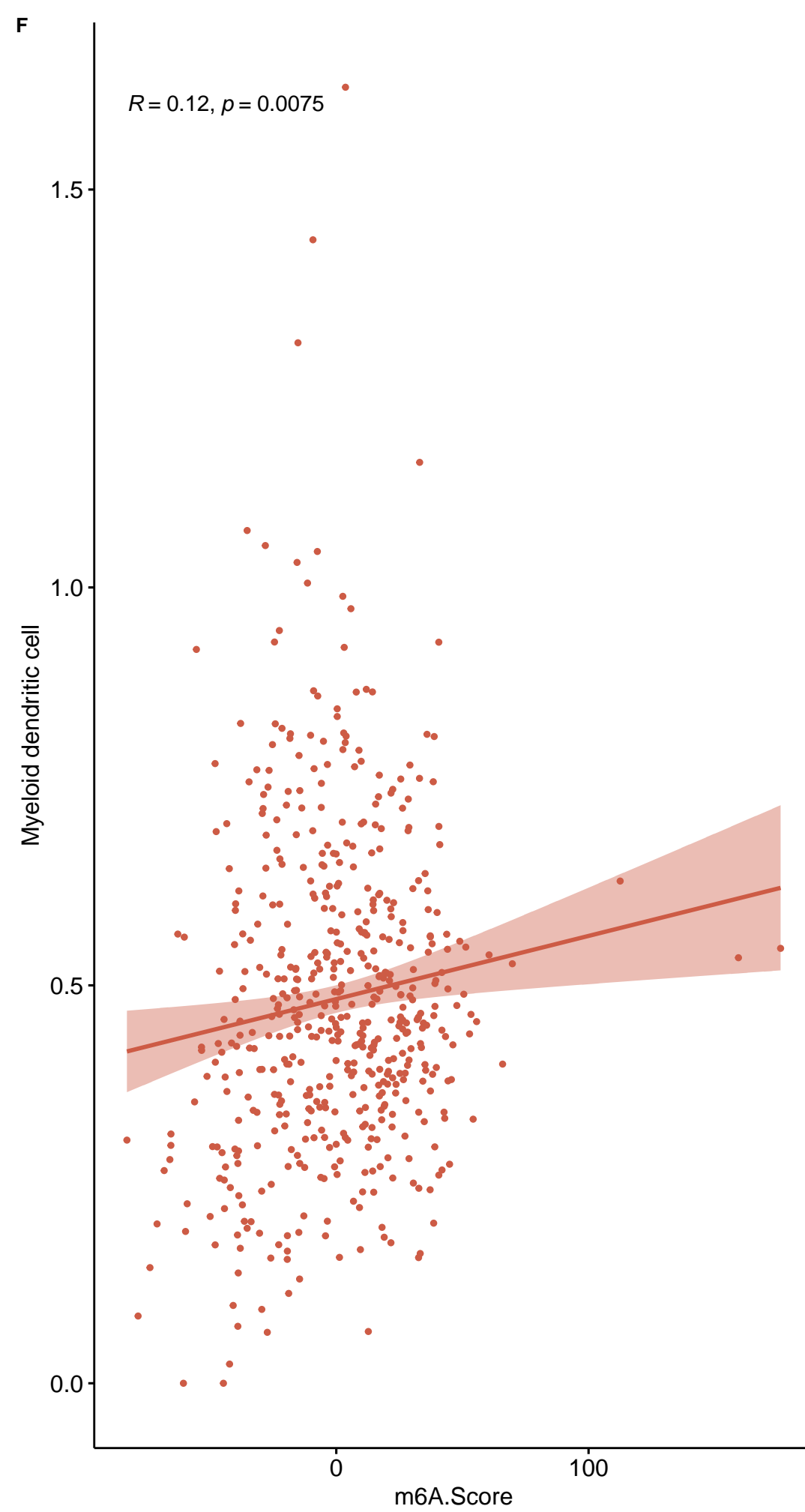

Supplement: Supplementary Figure 6 — Correlations between m6A score and tumor-infiltrating immune cells calculated with TIMER algorithm. [file Image_6.pdf]
